# Supplementary material for: Annotating RNA motifs in sequences and alignments
Source: Nucleic Acids Res. 2014 Dec 17;43(2):691–8. doi: 10.1093/nar/gku1327 (PMC4333381; doi:10.1093/nar/gku1327)
Supplement: SUPPLEMENTARY DATA [file supp_43_2_691__index.html]

Annotating RNA motifs in sequences and alignments — Annotating RNA motifs in sequences and alignments — SUPPLEMENTARY DATA 

# Annotating RNA motifs in sequences and alignments

## SUPPLEMENTARY DATA

**Files in this Data Supplement:**

- SUPPLEMENTARY DATA
- SUPPLEMENTARY DATA
