# Supplementary material for: Annotating RNA motifs in sequences and alignments
Source: Nucleic Acids Res. 2014 Dec 17;43(2):691–8. doi: 10.1093/nar/gku1327 (PMC4333381; doi:10.1093/nar/gku1327)
Supplement: SUPPLEMENTARY DATA [file supp_gku1327_nar-02481-r-2014-File002.pdf]

Supplementary results: Annotating RNA motifs in sequences and alignments

Paul P. Gardner[1,2]*, Hisham Eldai[1]

Abstract
This document contains the supplementary results for an article titled "Annotating RNA motifs in sequences and alignments".
It includes further details of the benchmarking tests of the annotation software as well as secondary structures for each of the motifs included in RMfam.
All the data and scripts associated with this work is available from: https://github.com/ppgardne/RMfam

Keywords
RNA — Motifs — Homology

[1] *School of Biological Sciences, University of Canterbury, Christchurch, New Zealand.*
[2] *Biomolecular Interaction Centre, University of Canterbury, Christchurch, New Zealand.*
***Corresponding author**: paul.gardner@canterbury.ac.nz

Summary

In the following document we present supplementary methods, results and figures relating to the RMfam resource:

1. Figures 1-8 illustrate secondary structure diagrams for each of the RMfam motifs. Figure 1 contains a Legend, detailing the color and symbol schemes used to illustrate different evolutionary constraints on the different structures.
2. Figure 9 illustrates our estimates of the accuracy of using covariance models to annotate RNA motifs on sequences and alignments.
3. Figures 10-43 contain secondary structures and the results of per-motif benchmarks.
4. Figures 44&45 illustrate improvements to Rfam (v11.0) alignments and consensus structures based upon RMfam annotations.
5. Figure 46 illustrates the network of the 50 highest scoring RMfam to Rfam mappings.

Benchmarking

In order to ensure that our approach provides accurate predictions we have carried out extensive benchmarking of the covariance models. These have been broken into three phases. We ran three different benchmark approaches *RMfam sequence benchmark*, *RMfam2Rfam alignment benchmark* and a *RMfam2Rfam sequence benchmark* on all the RMfam covariance models.

These benchmarks can be distinguished primarily by what is considered a *true positive*.

RMfam sequence benchmark

Unfortunately, most of the alignments in RMfam are composed of few sequences. In fact, the median number of sequences in the RMfam alignments is just 34.5. This means that idealised benchmarking strategies, such as cross-validation, are unlikely to provide useful results. Therefore we tested these covariance models on the training (seed) sequences, using a large negative control. This consisted of 10 permuted sequences for each seed sequence and 10 permuted sequences for each PDB sequence [1]. In order to control for sequence composition biases the di-nucleotide content was preserved between the native and permuted sequences [2]. Also, in order to identify members of the motif family with solved structures, we ran the CMs over 11,508 nucleotide sequences extracted from the June 2014 release of PDB.

We used the results of this benchmark to identify a bit score threshold, this value ideally discriminates between the true members of the family and the negative control (permuted) sequences. In practise, a slightly lower than optimal threshold is generally selected as *false positives* are generally considered to be more desirable than *false negatives*.

The results of these tests are illustrated in Supplementary Figures 10-43.

RMfam2Rfam alignment benchmark

There are many instances of RNA families (Rfam) with good evidence that they host RNA motifs. Many of these have been published in the literature. For the purposes of benchmarking we have curated a collection of motifs in Rfam, including annotating the evidence associated with these (See Supplementary Table 1), the bulk (261/446) of these are derived from Cruz and Westhof (2011) [3], 37 are from other publications [4, 5, 6, 7, 8, 9, 10, 11, 12, 13, 14, 15, 16, 3, 17, 18, 19] and

148 were curated by ourselves. These connections between RMfam and Rfam cover 238/2208 Rfam families and 21/34 RMfam motifs.

In order to automate the prediction of motifs in Rfam alignments we built a Perl wrapper (rmfam_scan.pl), which is available on GitHub: `http://github.com/ppgardne/RMfam`. Our approach begins by making the input Rfam (version 11.0) seed alignments non-redundant by filtering out sequences that are more than 90% identity to each other. This threshold was selected as most (87%) of the Rfam (v10.1) alignments have sequences more divergent than 90% similar and this threshold is well above the identity limit where the accuracy of RNA sequence alignment falls [20, 21].

We annotate the remaining sequences with each RMfam motif, using the score threshold determined during the "RMfam sequence benchmark". We further filter these annotations by selecting only those that are identified in two or more **and** $\geq$ 10% of the sequences in each Rfam alignment.

We experimented with a number of approaches for generating negative control alignments that preserved the characteristics of sequence conservation found in the Rfam alignments, including multiperm [22], SISSIz [23], "esl-shuffle" [24] and "shuffle-aln.pl" from the RNAz package [25]. We selected shuffle-aln.pl for generating our negative controls because it (A) ran on our computers and (B) did not significantly alter key characteristics of the alignments e.g. sequence lengths and sequence identity (data not shown).

We experimented with a number of summary statistics for identifying "good" matches between our motifs and Rfam. These included the fraction of annotated sequences, a tree weighted sum of bit scores [26] and summing all bit scores for each motif in each Rfam alignment (See Supplementary Figure 9). We selected the latter (sum of bit scores) as the preferred summary statistic, as this provided the maximum Matthew's Correlation Coefficient (MCC) of all the measures we tested and is trivial to compute (Figure 9).

RMfam2Rfam sequence benchmark

The depths of Rfam seed alignments can vary from 2 to 1,020 sequences. Consequently, measures like sum-of-bits can be a reflection of the numbers of sequences in alignments rather than the likelihood that they host a motif. In order to compensate for this we sampled up to 5 sequences from each Rfam seed alignment, and ran a sequence annotation over these sequences (skipping the similarity reduction and the minimum number of sequences filters used for the alignment benchmark). Ten shuffled versions of each sampled Rfam sequence were also generated and annotated.

Definitions of performance measures

In the following results we display a range of performance measures for all RMfam annotations. We briefly summarize these below. Each prediction is classified as either a *true positive* (TP), *true negative* (TN), *false positive* (FP) or *false negative* (FN).

The totals of these can be used to compute a range of performance statistics. These include the *Sensitivity* or fraction true data that are correctly assigned, the *Specificity* or the fraction of false data that are correctly assigned, the *Positive Predictive Value* (PPV) or the fraction of predicted trues that are correct, the *Negative Predictive Value* (NPV) or the fraction of false predictions that are correct, the *False Discovery Rate* (FDR) or the fraction of true predictions that are incorrect, the *Accuracy* (ACC) or the fraction of all predictions (true and false) that are correct, the *False Positive Rate* (FPR) or the fraction of false predictions that are actually false.

Finally, a common measure for determining the accuracy of a method is to compute the *Matthew's Correlation Coefficient* (MCC). This measure ranges between +1 and −1, a value of +1 indicates a perfect discrimination between true and false members, a value of 0 implies no predictive power and a value −1 indicates a completely imperfect discrimination between true and false positives.

$$Sensitivity = \frac{TP}{TP+FN} \qquad Specificity = \frac{TN}{FP+TN}$$

$$PPV = \frac{TP}{TP+FP} \qquad NPV = \frac{TN}{TN+FN}$$

$$FDR = \frac{FP}{TP+FP} \qquad FPR = \frac{FP}{FP+TN}$$

$$MCC = \frac{TP \times TN - FP \times FN}{\sqrt{(TP+FP)(TP+FN)(TN+FP)(TN+FN)}}$$

$$ACC = \frac{TP+TN}{TP+TN+FP+FN}$$

Individual motif performance

The following figures (S10 to S43) illustrate the annotation accuracy for each of the motifs in RMfam. On the far **left** of each figure is an illustration of the motif secondary structure and sequence conservation, see Figure 1 for a legend. In the **middle** is an illustration of the covariance model score distributions over sequences derived from the PDB, sequences from the RMfam seed alignments and shuffled PDB and RMfam counterparts. A curated "threshold", for distinguishing between true and false sequence matches is illustrated with a dashed vertical line. The **right** figure contains four panels, starting from the top-left and moving around the plot in a clockwise direction, these are: ROC-curves for each of the 3 benchmarks described previously; ROC-like-curves, of PPV

vs Specificity; a bar plot illustrating the MCC, sensitivity (SENS), specificity (SPEC), positive predictive value (PPV), negative predictive value (NPV), accuracy (ACC) and the false discovery rate (FDR), each of these was computed using the threshold that maximises the MCC; The MCC shown as a function of the covariance model bitscore (or sum of bit scores in the alignment benchmark).

Secondary structures

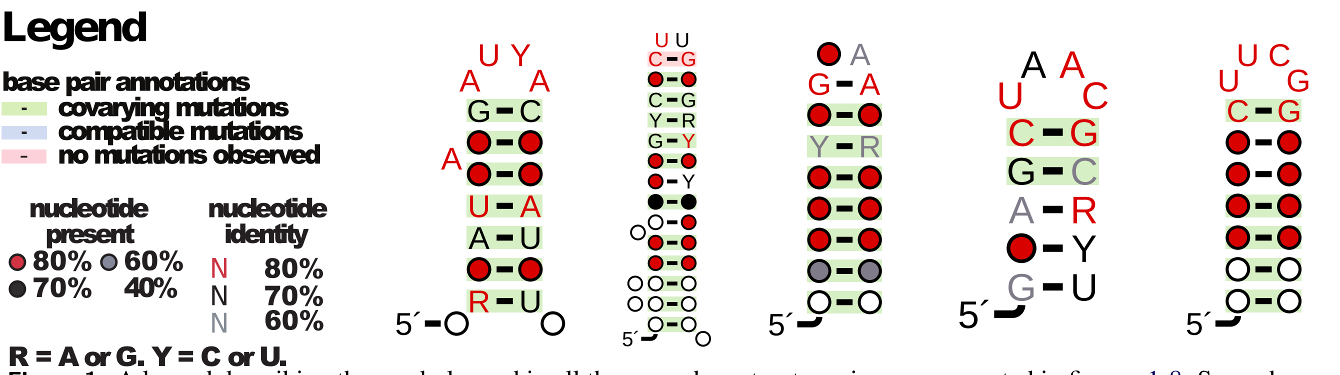

Figure 1. A legend describing the symbols used in all the secondary structures images presented in figures 1-8. Secondary structure diagrams of: tetraloops: ANYA [27, 28, 29], CUYG [30, 31, 32, 33], GNRA [34, 35, 36, 37, 38, 39], UMAC [40, 41] and UNCG [38, 39, 42, 36] and the hairpins loops C-loop [17, 43, 44, 3], T-loop [38, 39, 45, 10, 46] and U-turn [38, 39, 7, 47].

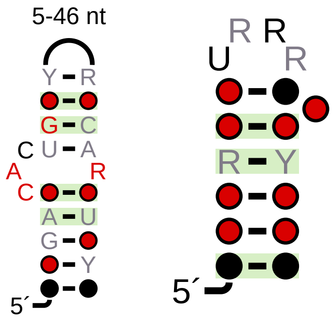

Figure 2. Secondary structure diagrams of: the hairpins loops; C-loop [17, 43, 44, 3], T-loop [38, 39, 45, 10, 46] and U-turn [38, 39, 7, 47].

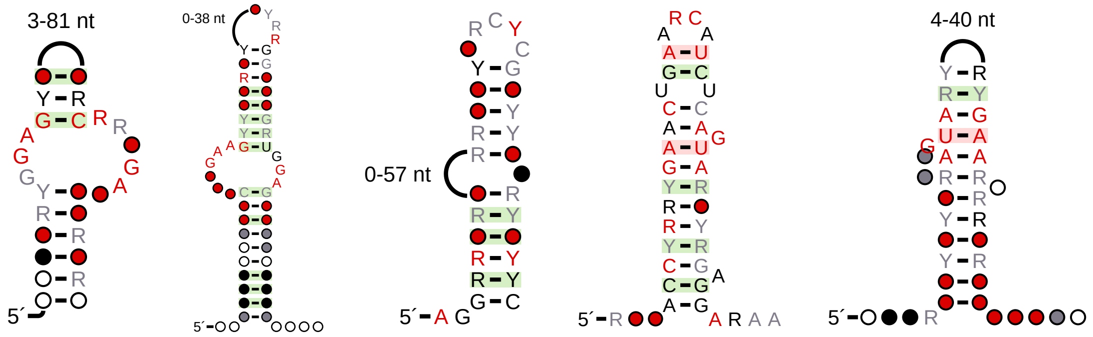

Figure 3. Secondary structure diagrams of: internal loops: three k-turns [29, 43, 38, 39, 15, 48, 49] and two sarcin-ricin loops [50, 51, 38, 3].

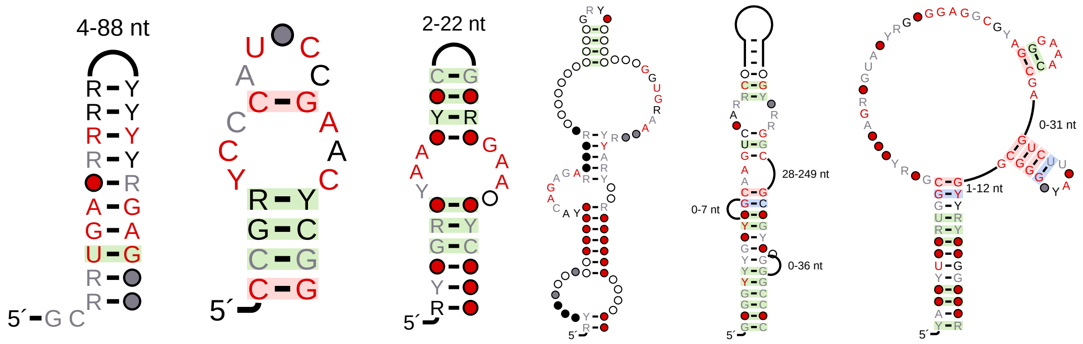

Figure 4. Secondary structure diagrams of: internal loops: the tandem-GA [52, 3], twist_up [17] and UAA_GAN [53], the docking elbow [19], right angle 2 and 3 [18] motifs.

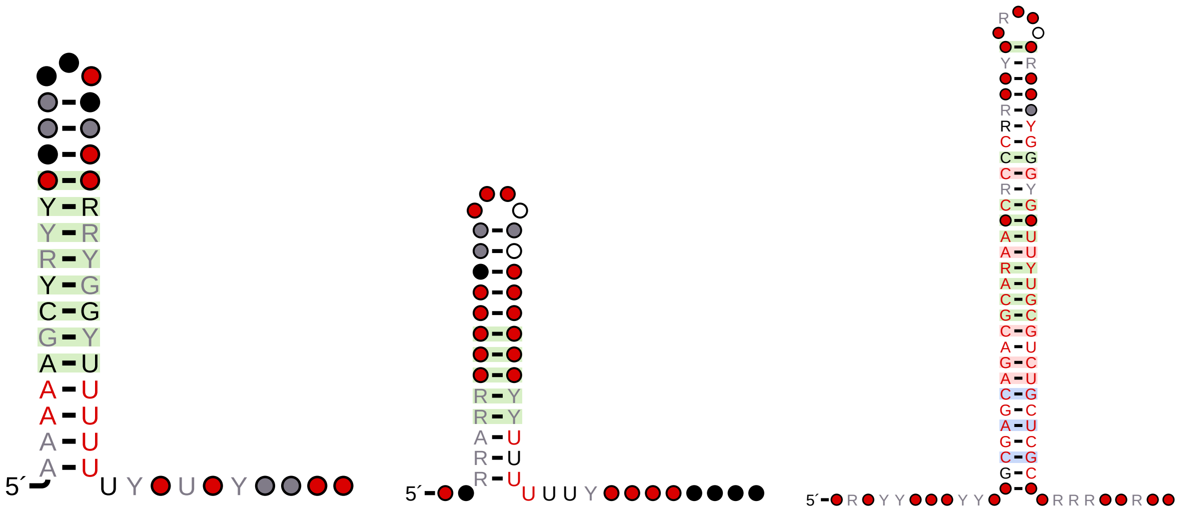

Figure 5. Secondary structure diagrams of Rho independent transcription terminators [54].

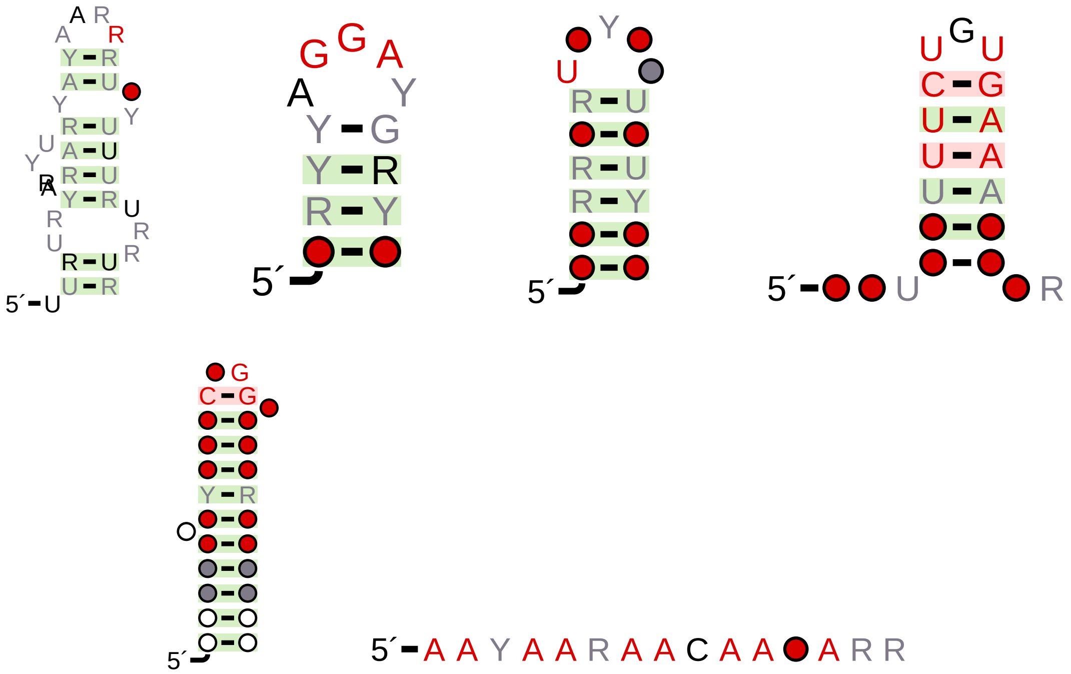

Figure 6. Secondary structure diagrams of: interactions: the AUF1 [55], CsrA [56, 57, 58, 59, 60, 61, 62, 63], HuR [64, 65], Roquin [66], VTS1 [67, 68, 69, 70] and CRC [13, 71, 72] protein binding motifs.

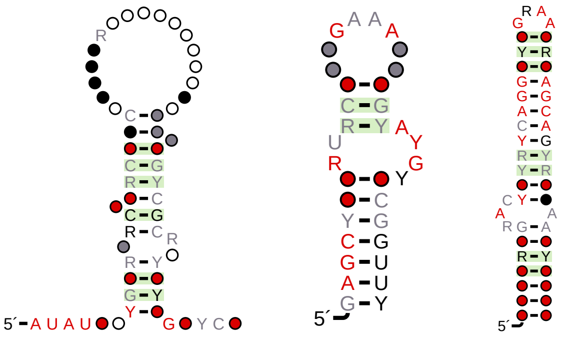

Figure 7. Secondary structure diagrams of: vapC target [73], the SRP RNA S domain [74, 75, 76] and the catalytic Domain-V [77, 78].

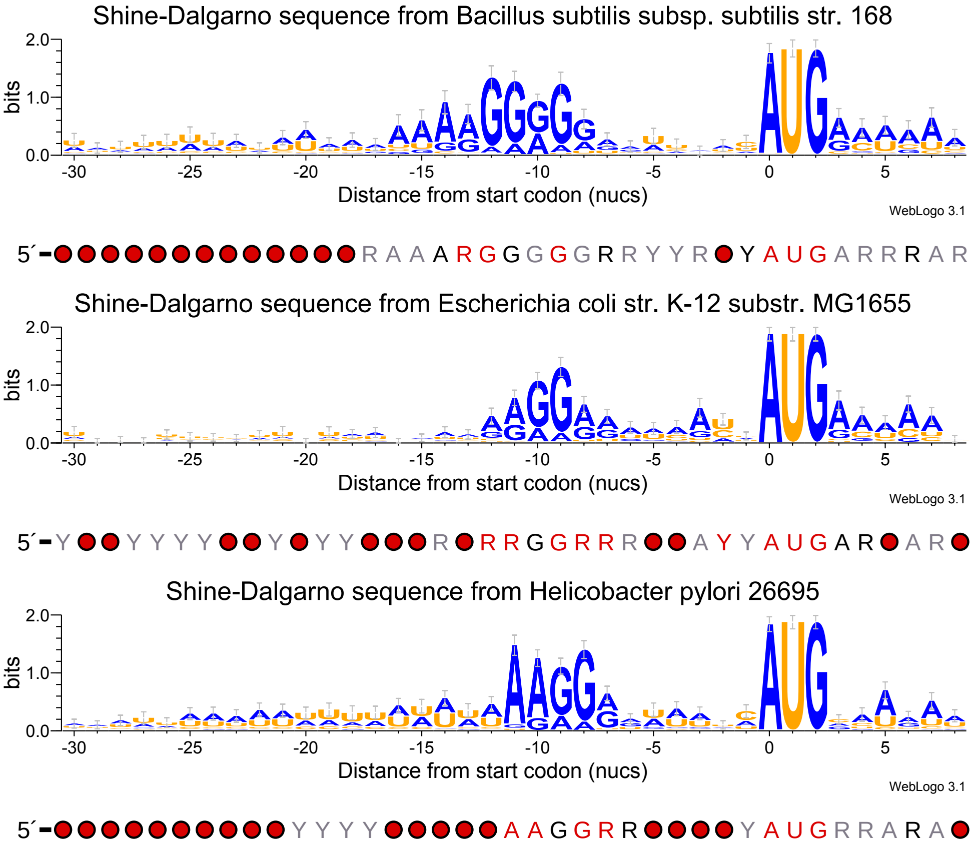

Figure 8. Secondary structure diagrams of: sequence motifs: Shine-Dalgarno sequences from *Bacillus subtilis*, *Escherichia coli* and *Helicobacter pylori* respectively [79].

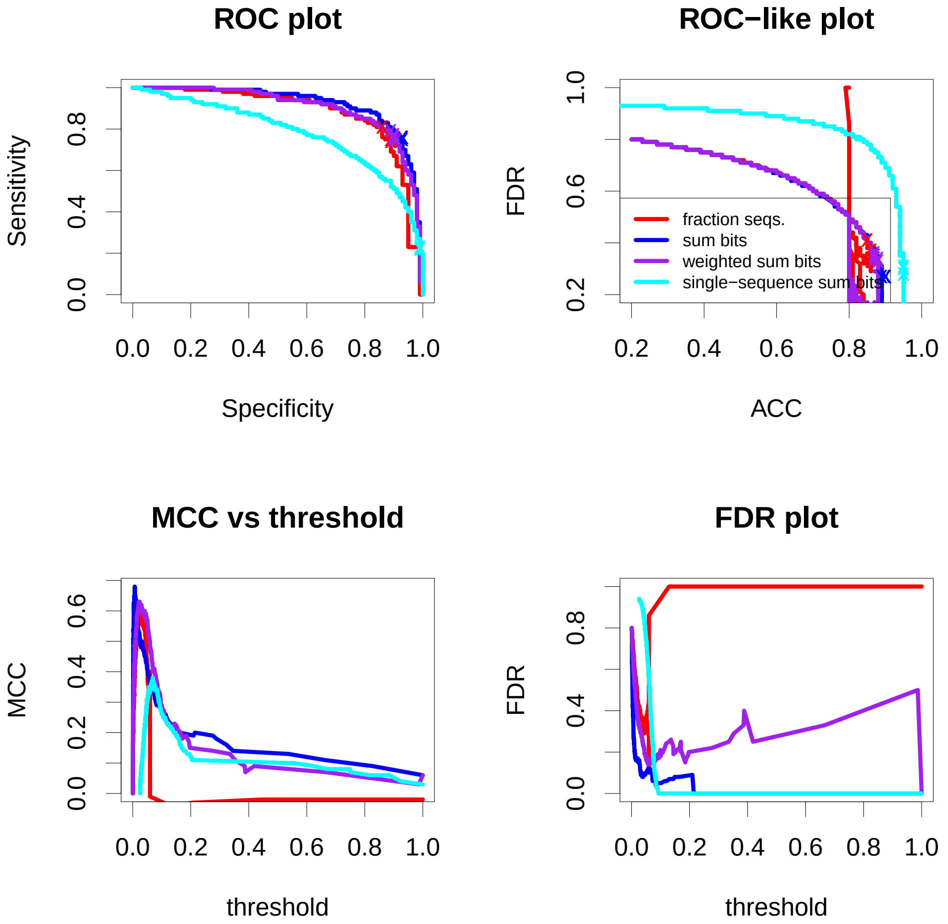

Figure 9. Testing a variety of summary statistics for identifying RMfam motifs in Rfam seed alignments. These were fraction of sequences, the sum of bit scores, a tree-weighted sum of bit scores and a sum of bit scores for single sequences sampled from each Rfam alignment. The top left figure is a ROC plot [80], the top right shows the false discovery rate versus accuracy trajectory for each score, the bottom left shows the Matthew's Correlation Coefficient

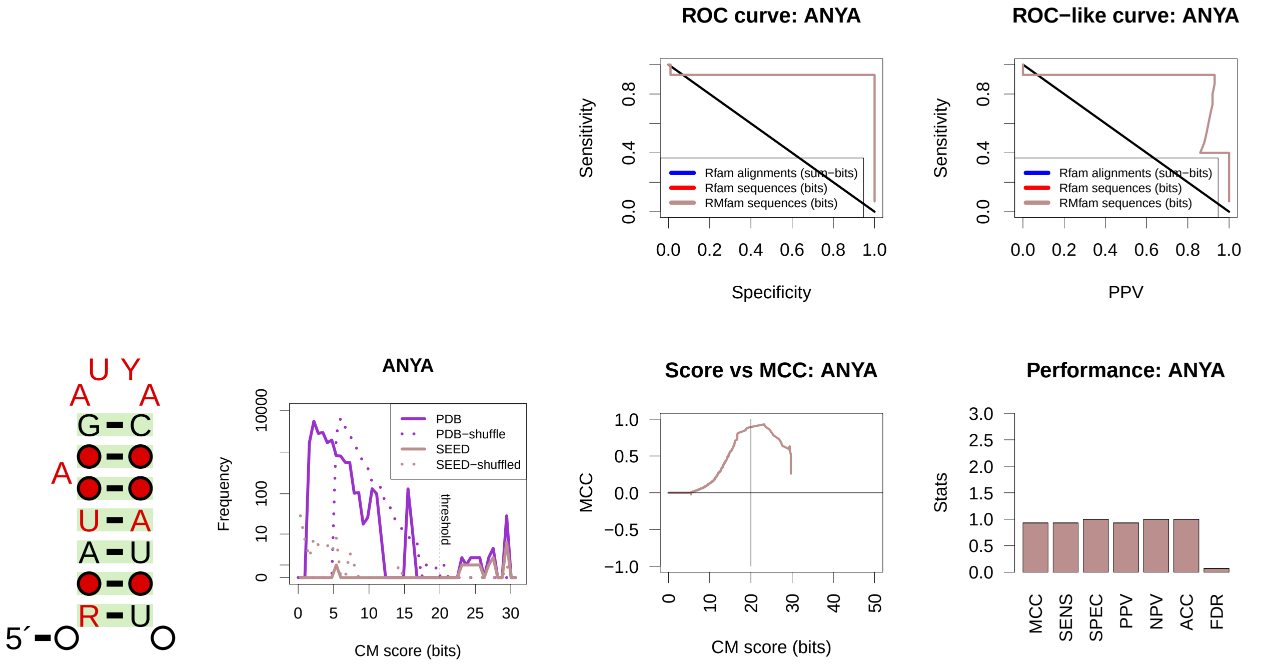

Figure 10. ANYA.

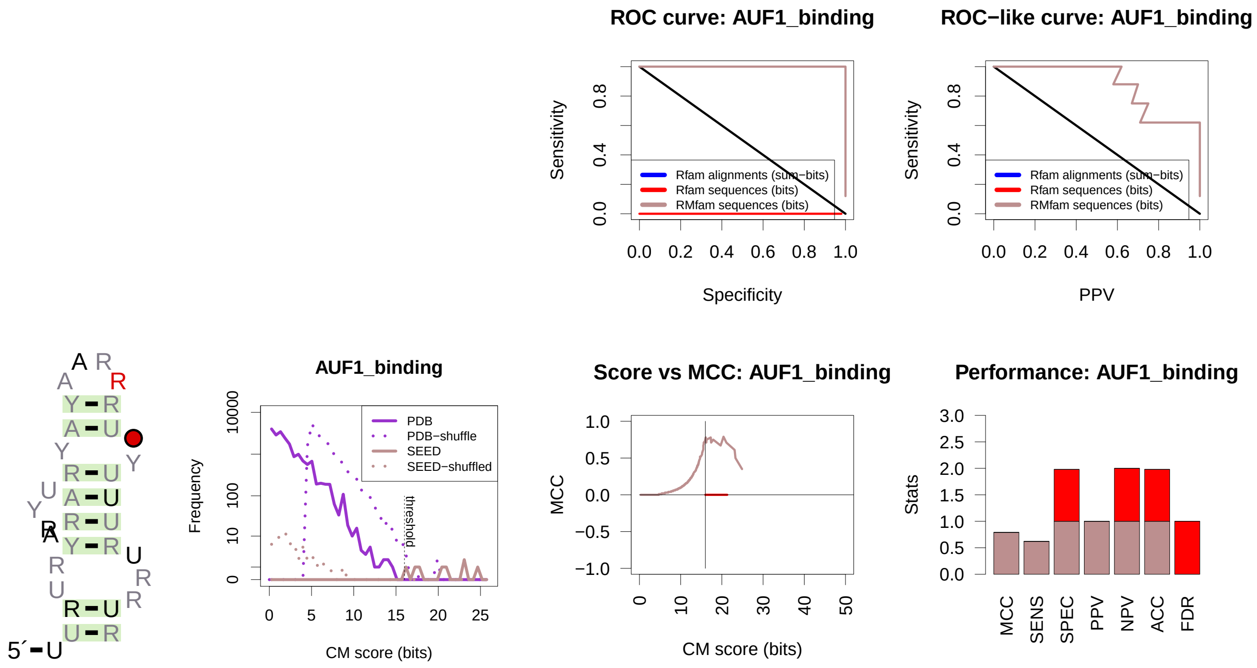

Figure 11. AUF1_binding.

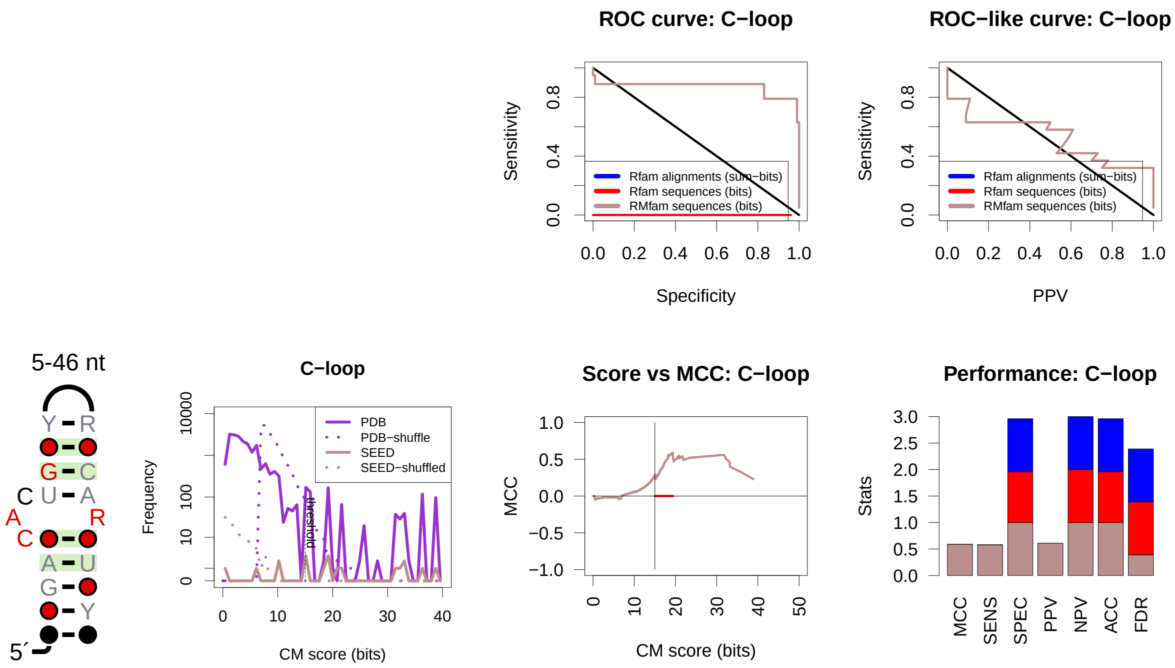

Figure 12. C-loop.

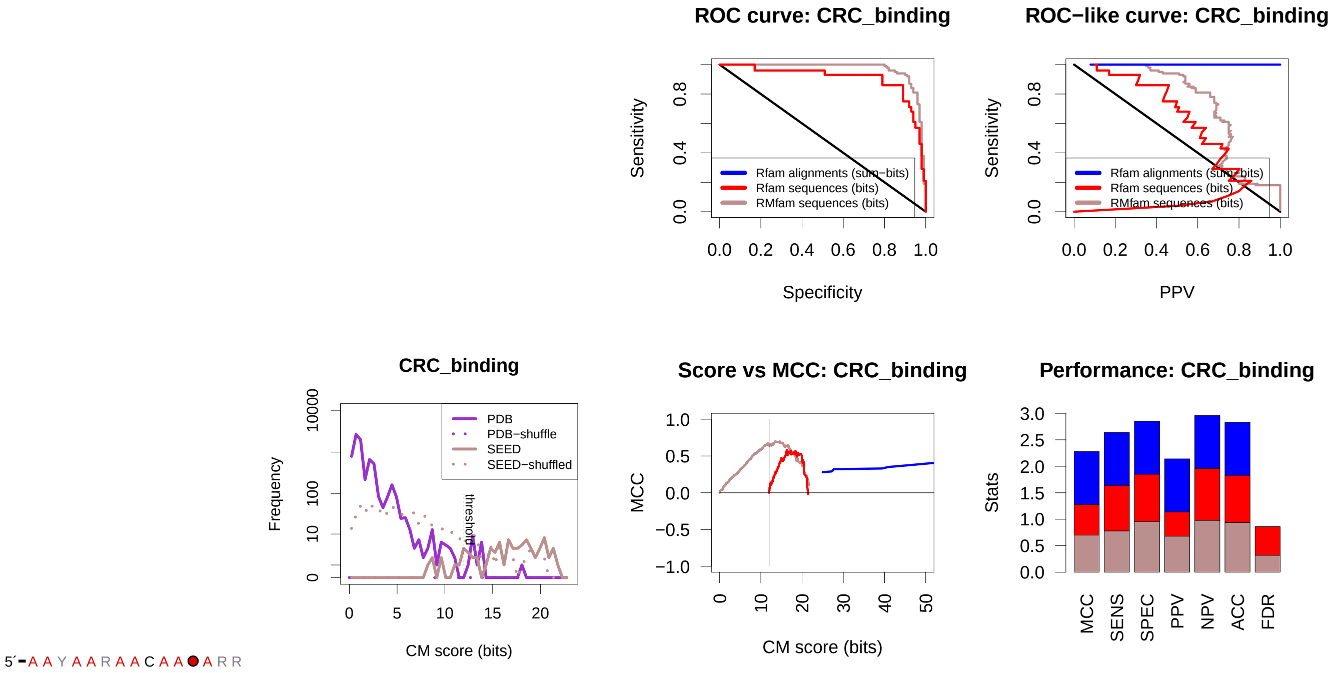

Figure 13. CRC_binding.

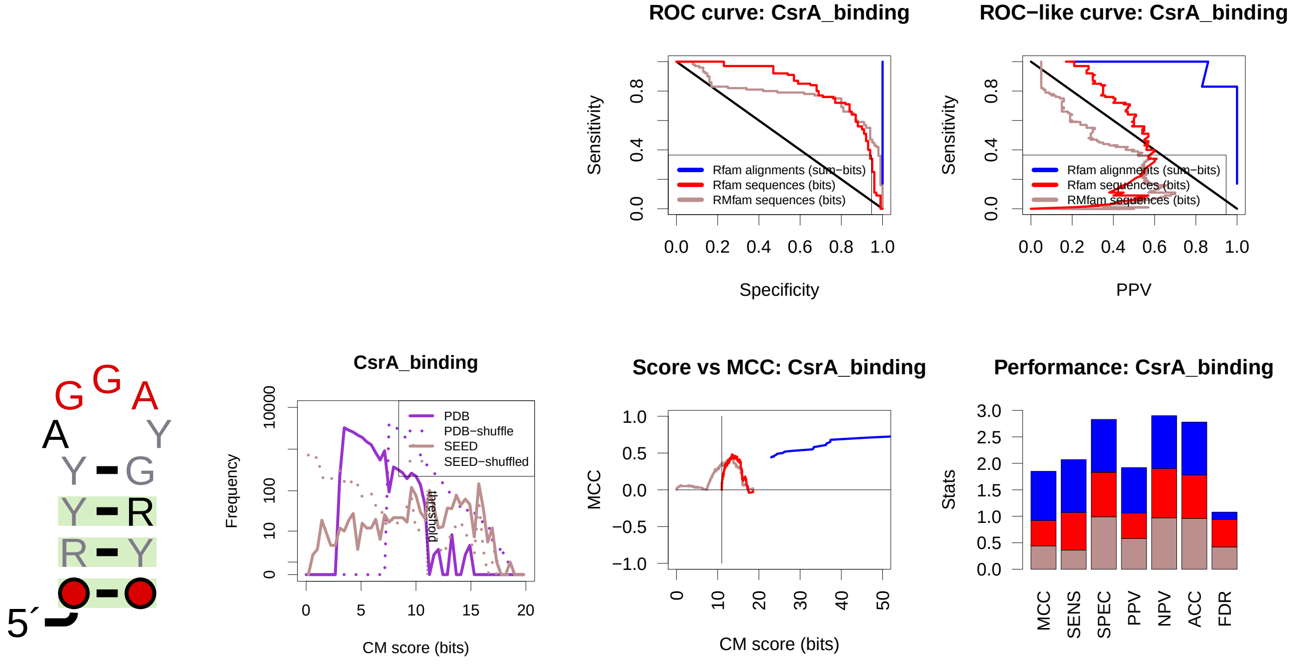

Figure 14. CsrA_binding.

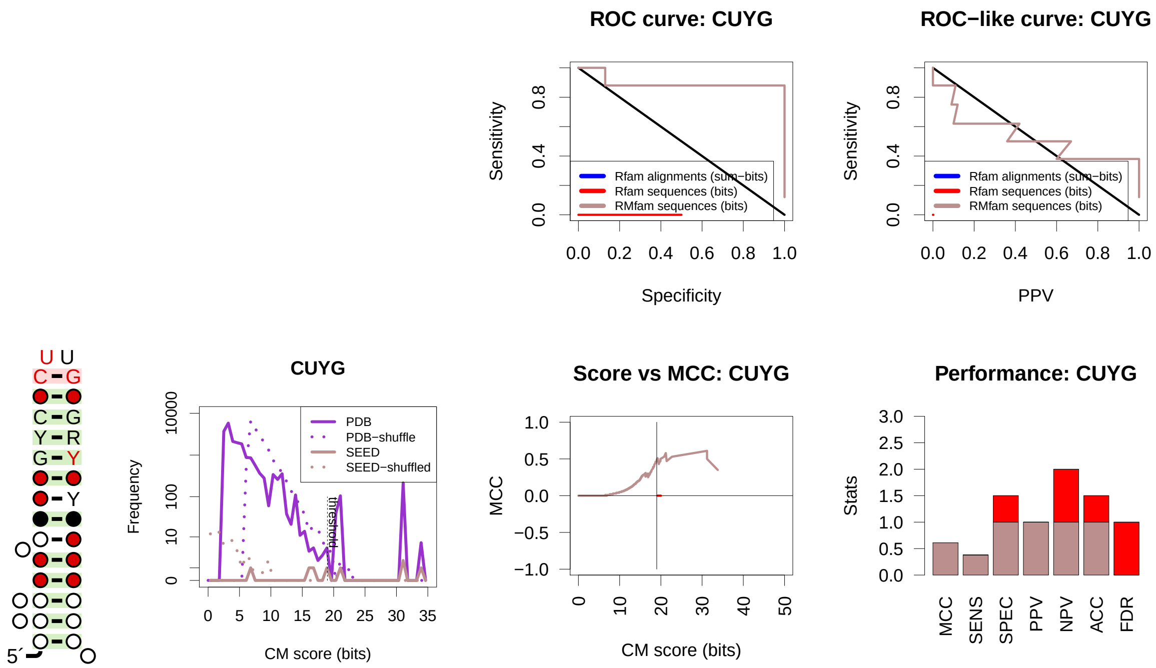

Figure 15. CUYG.

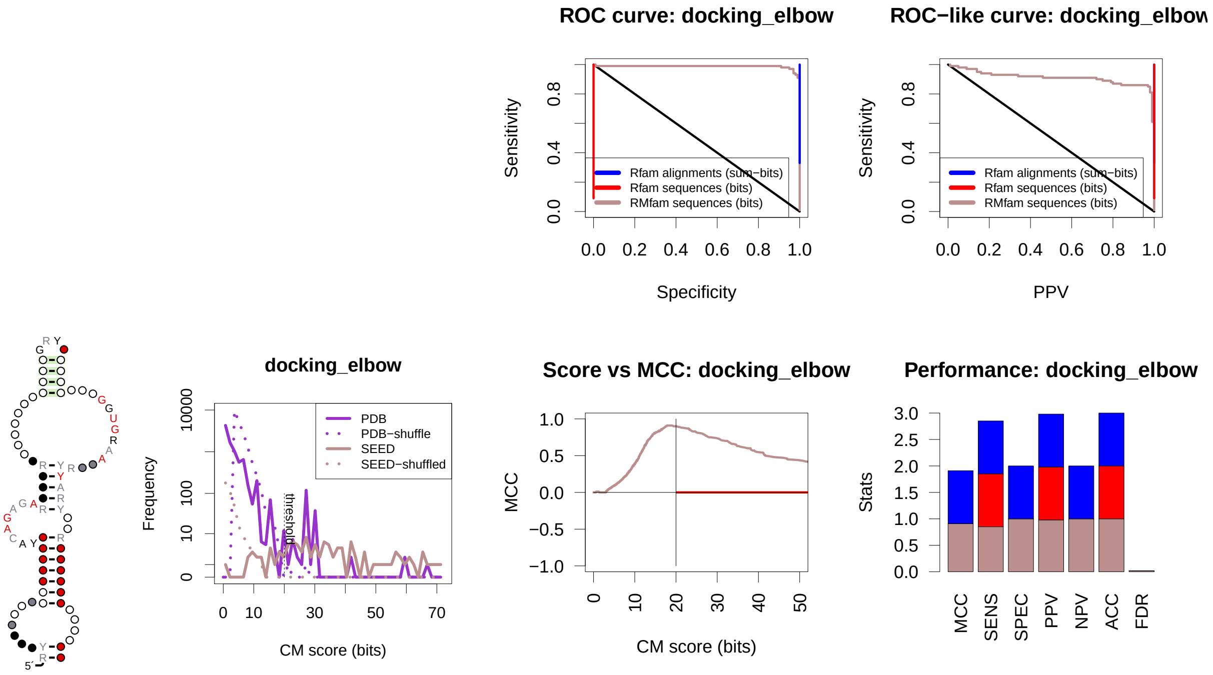

Figure 16. docking_elbow.

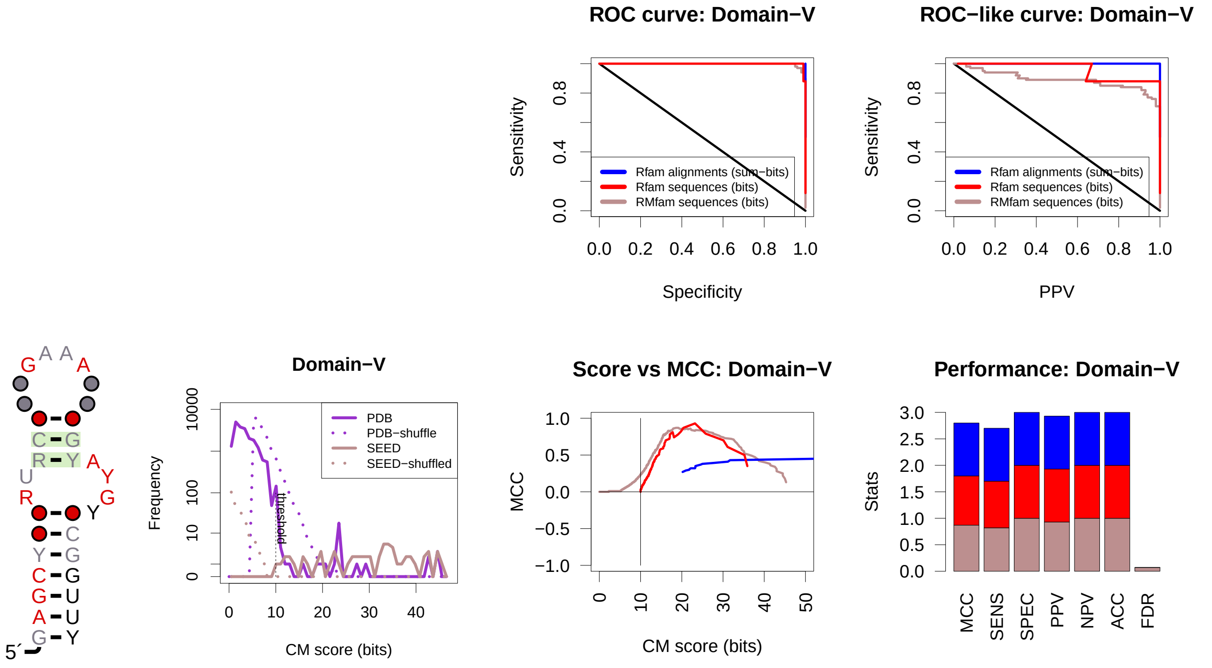

Figure 17. Domain-V.

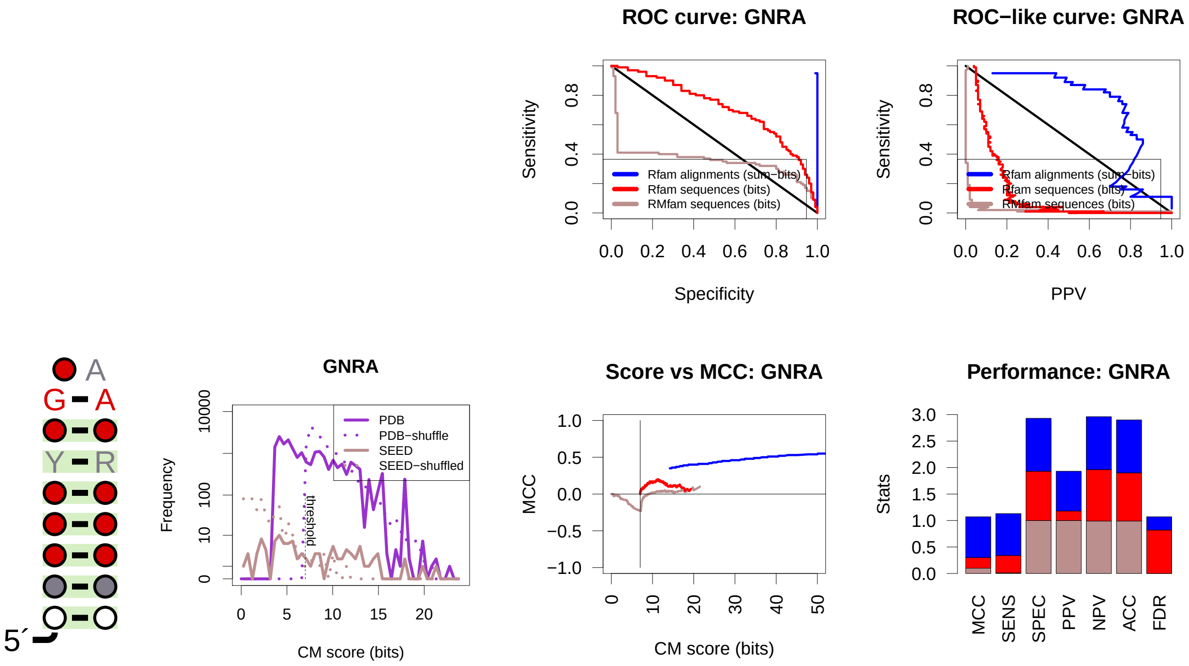

Figure 18. GNRA.

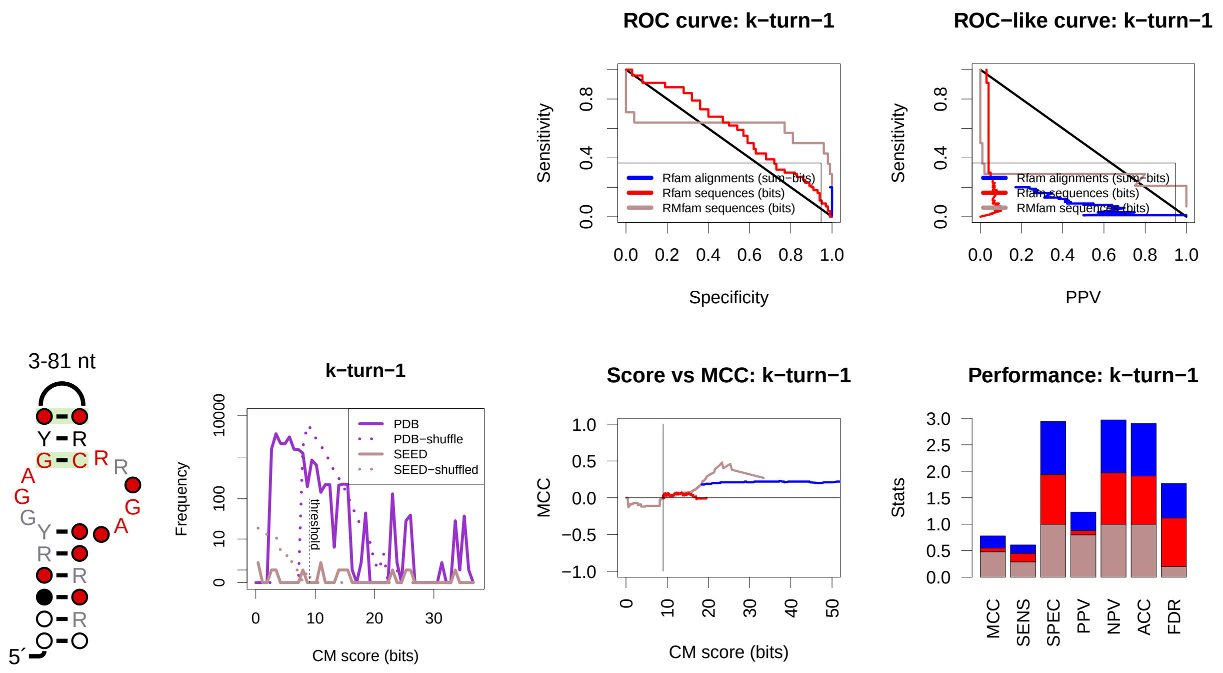

Figure 19. k-turn-1.

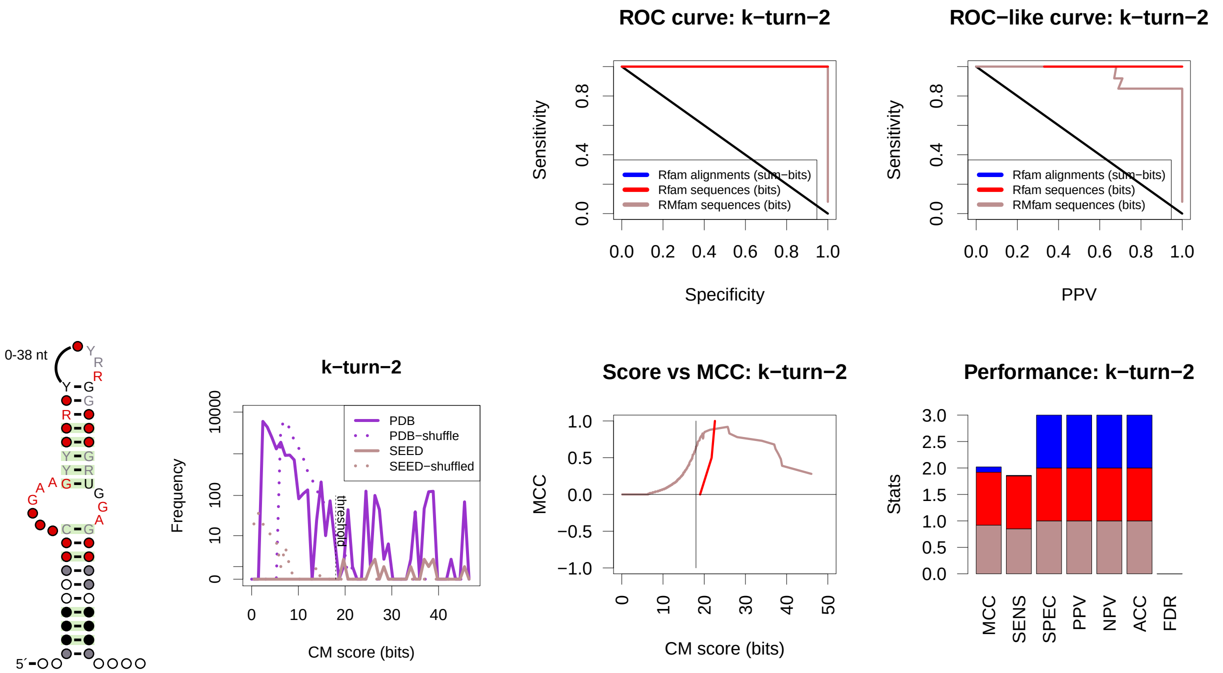

Figure 20. k-turn-2.

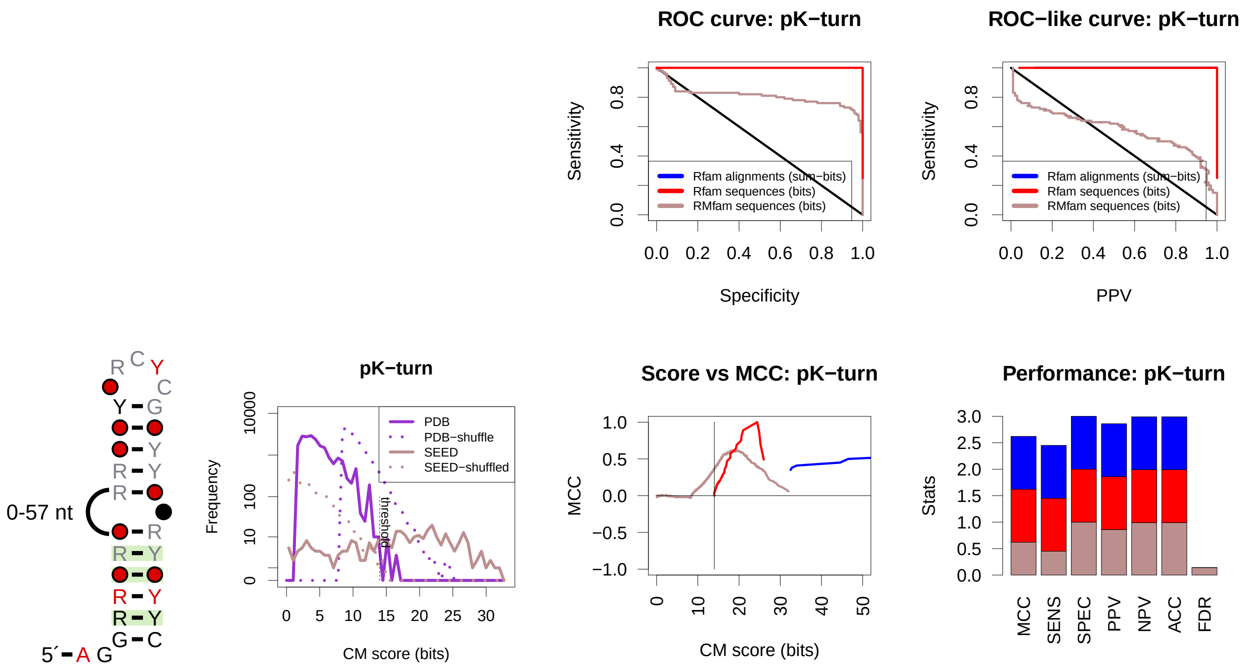

Figure 21. pK-turn.

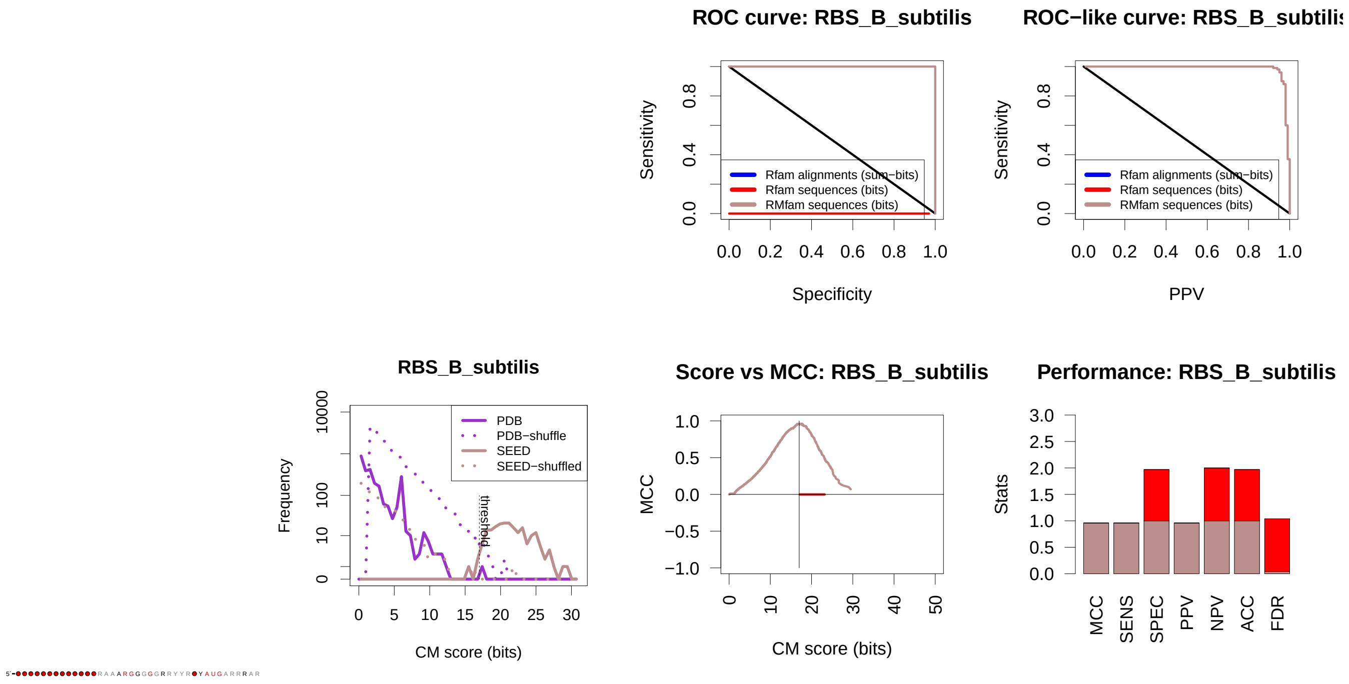

Figure 22. RBS_B_subtilis.

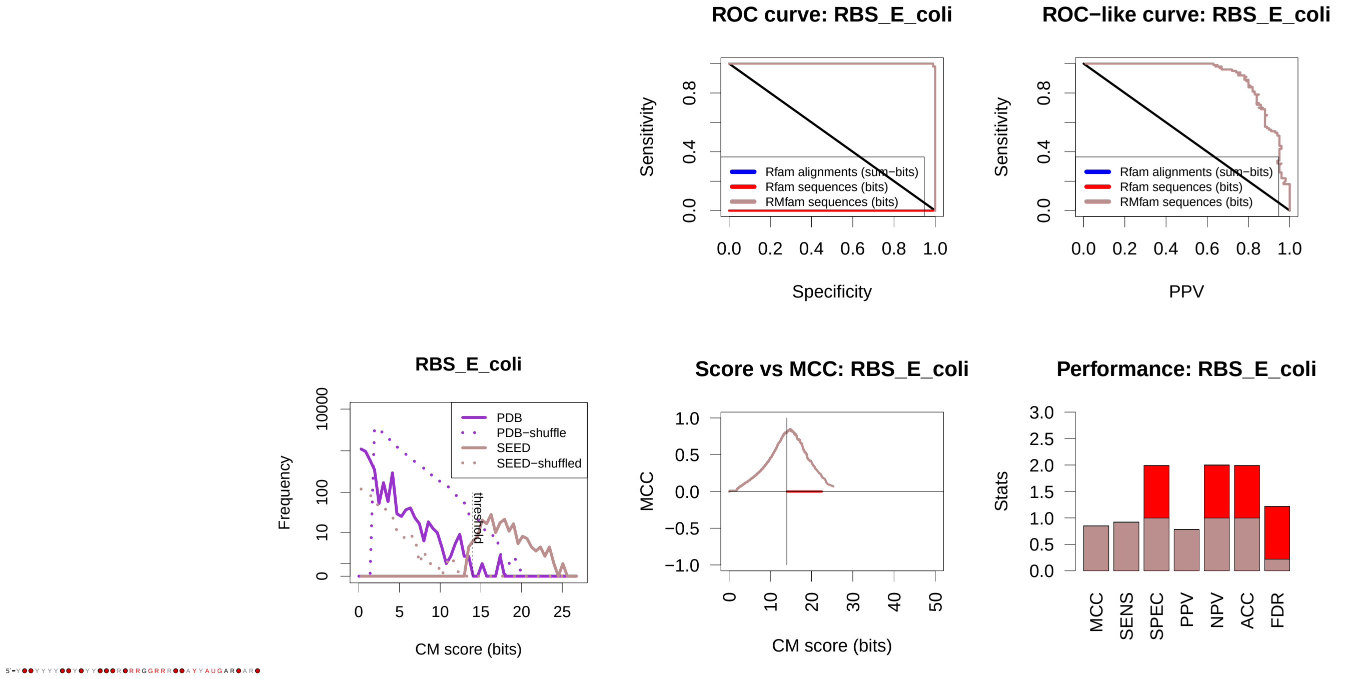

Figure 23. RBS_E_coli.

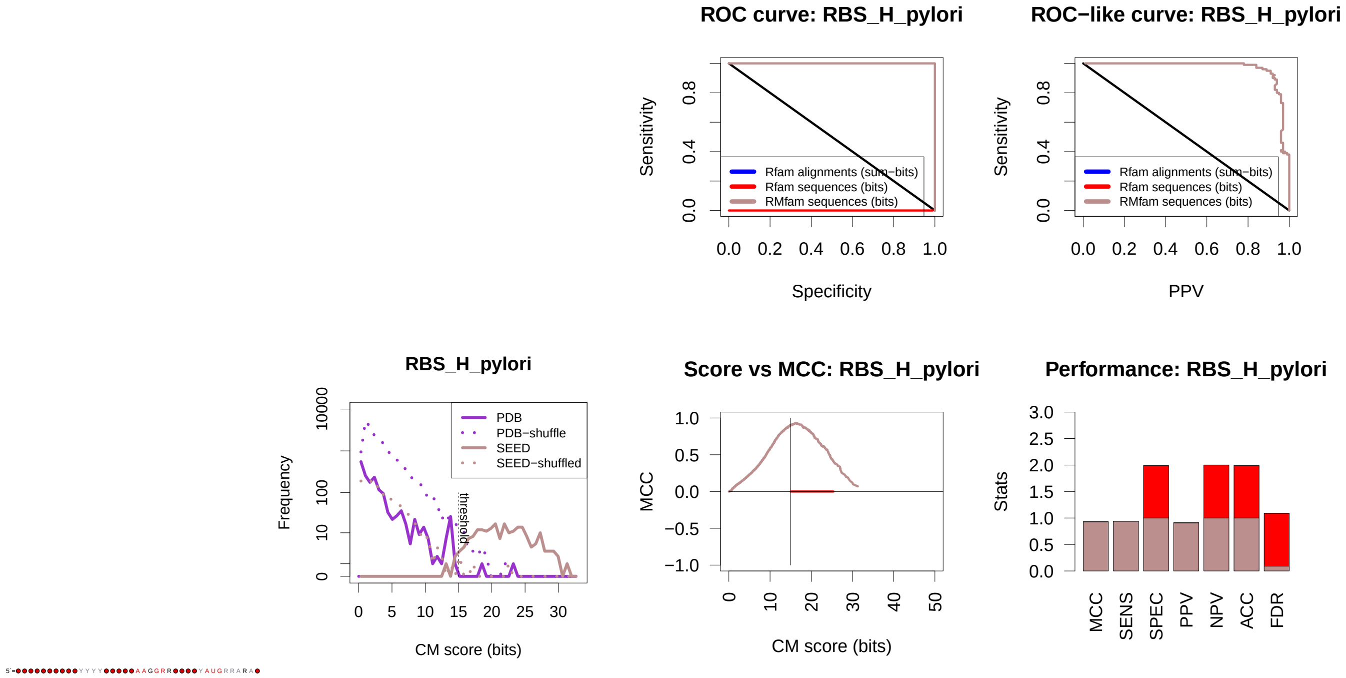

Figure 24. RBS_H_pylori.

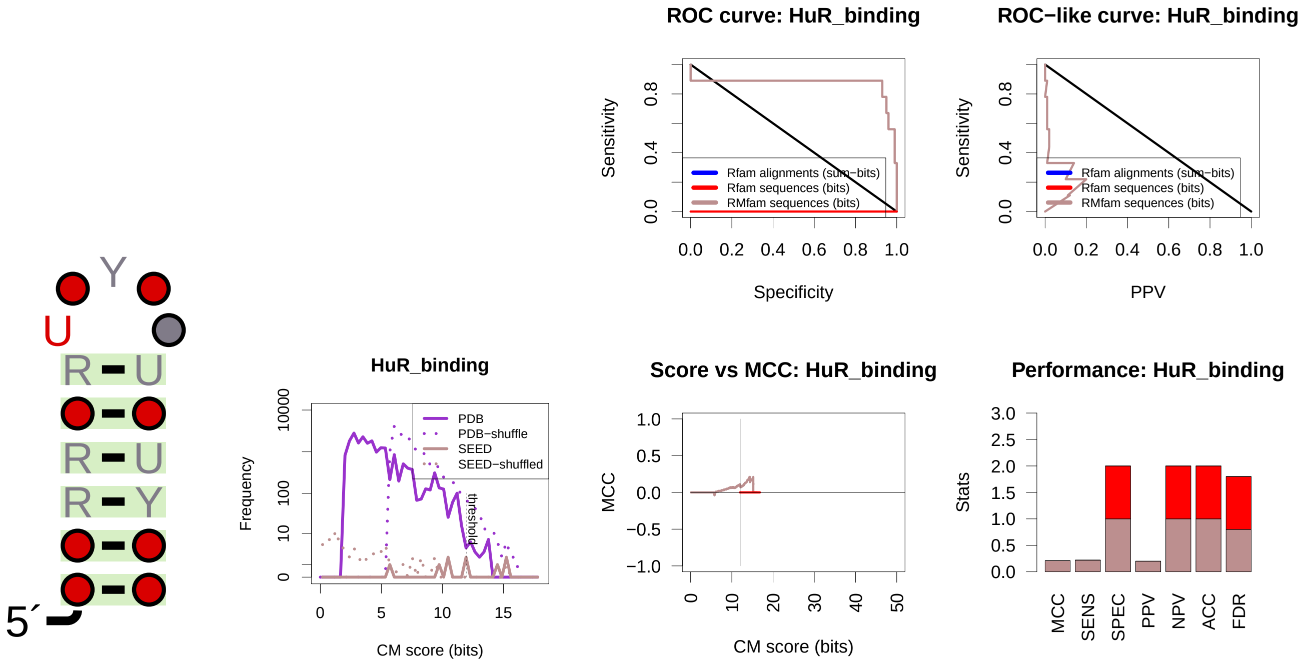

Figure 25. HuR_binding.

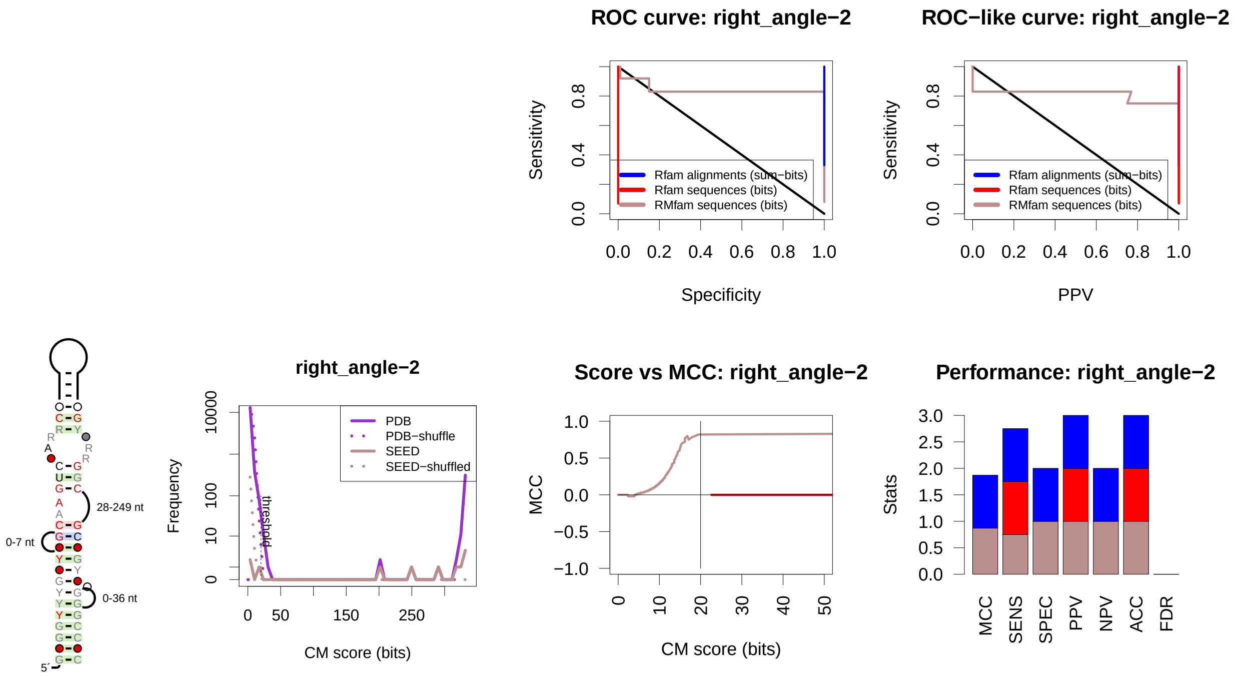

Figure 26. right_angle-2.

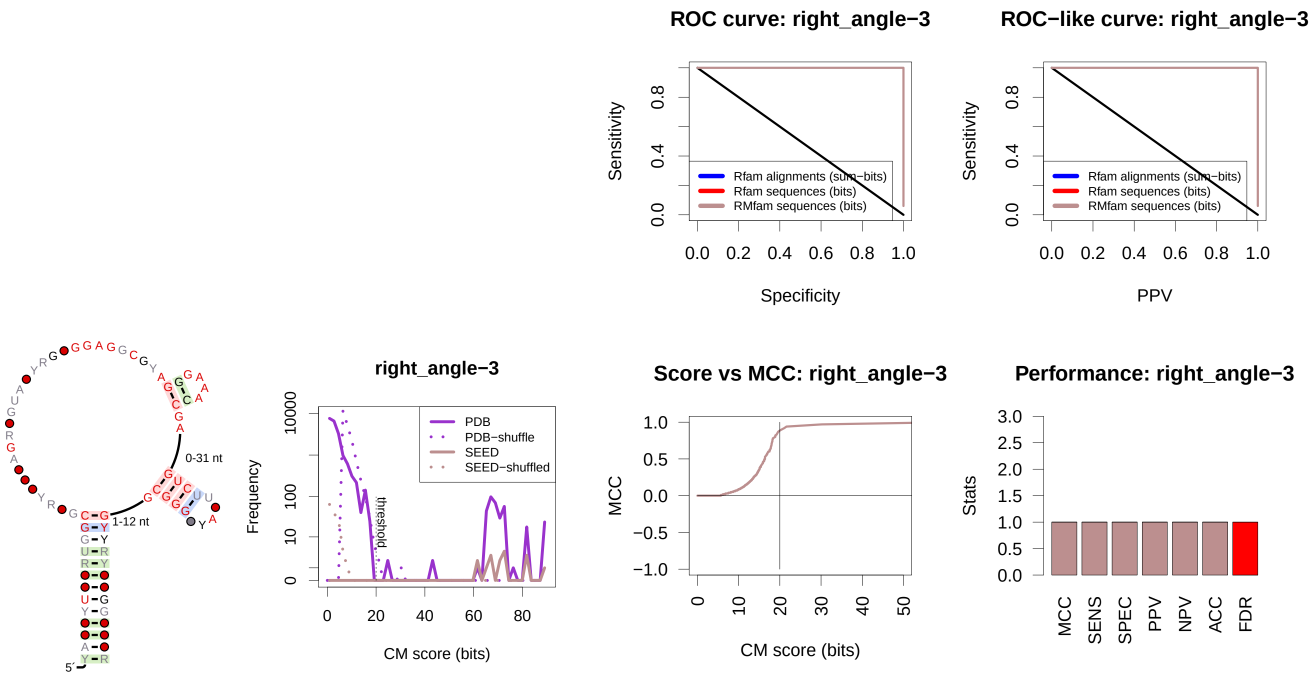

Figure 27. right_angle-3.

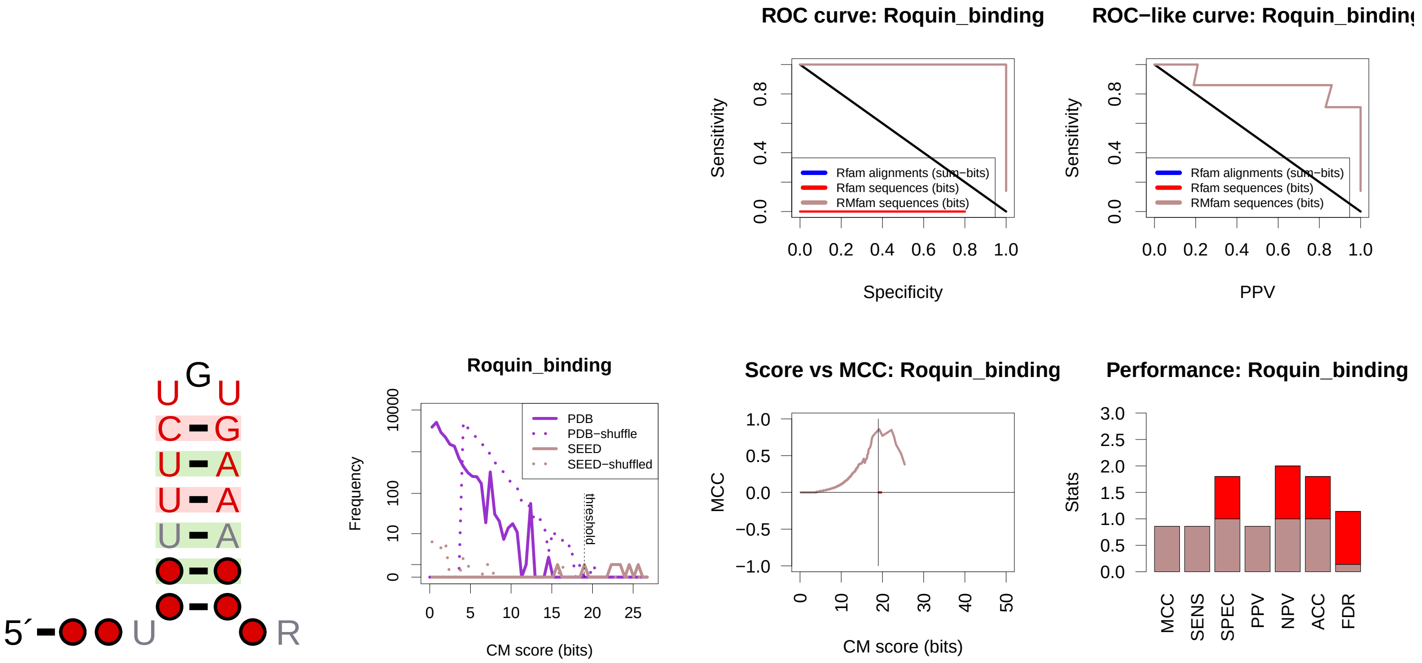

Figure 28. Roquin_binding.

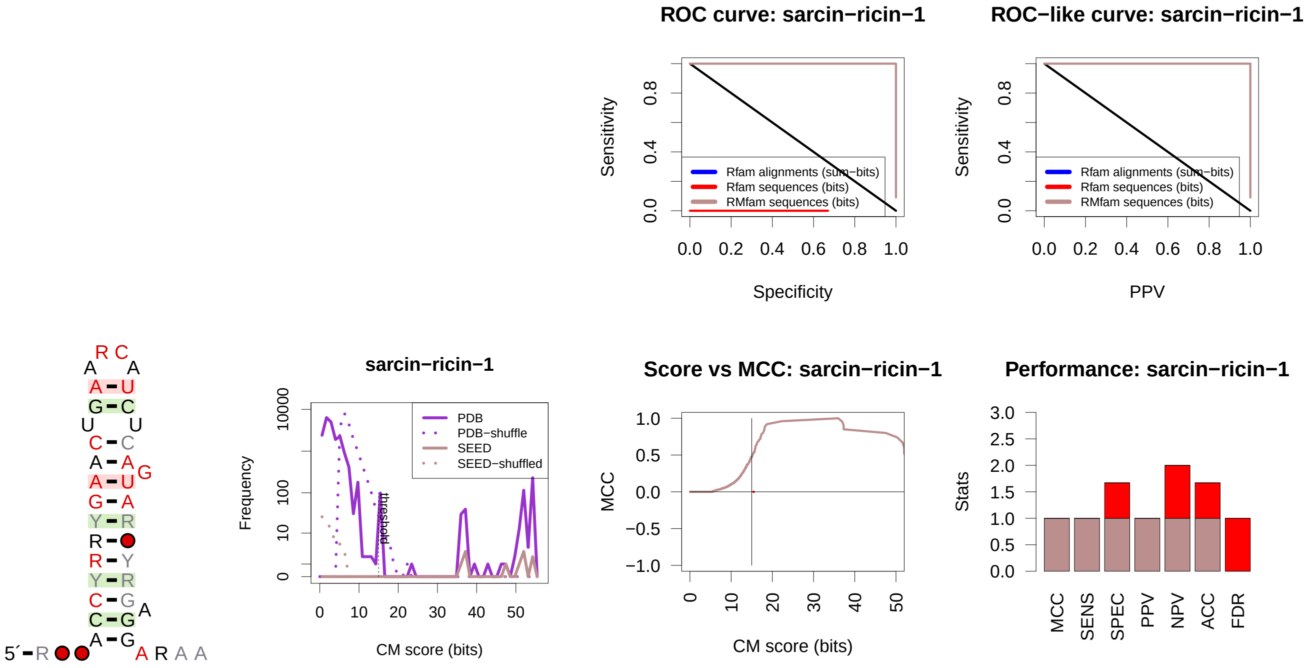

Figure 29. sarcin-ricin-1.

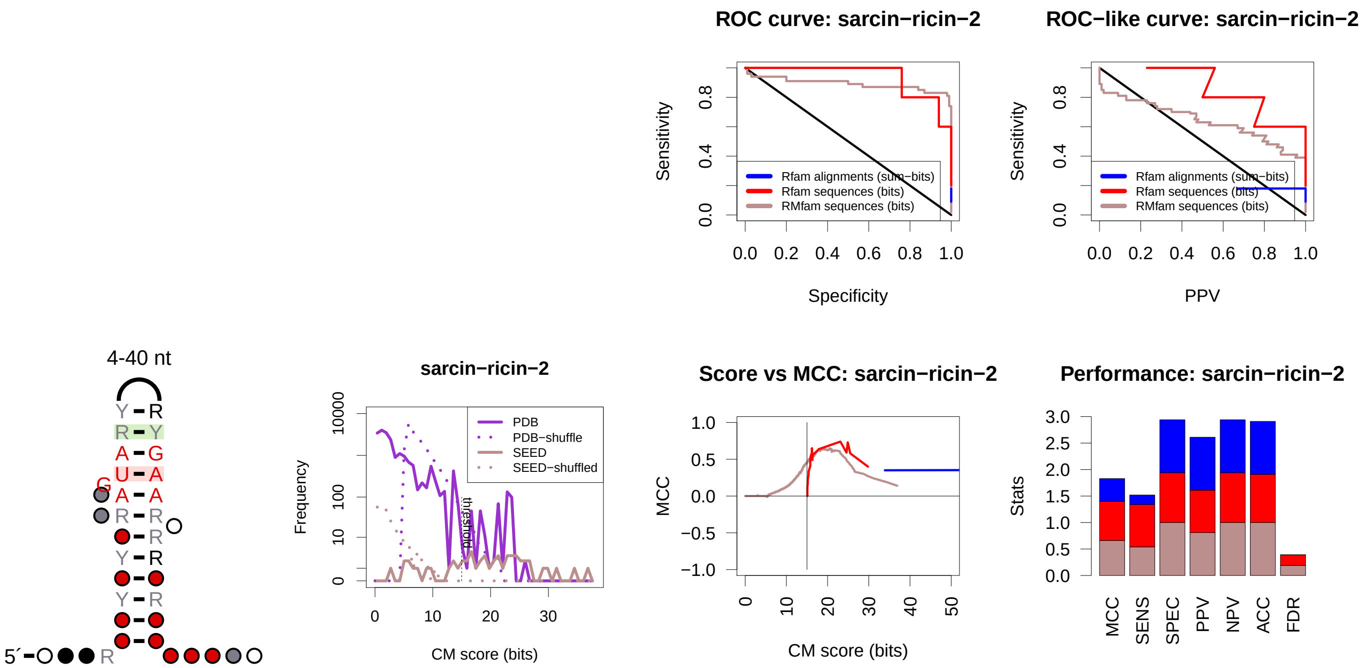

Figure 30. sarcin-ricin-2.

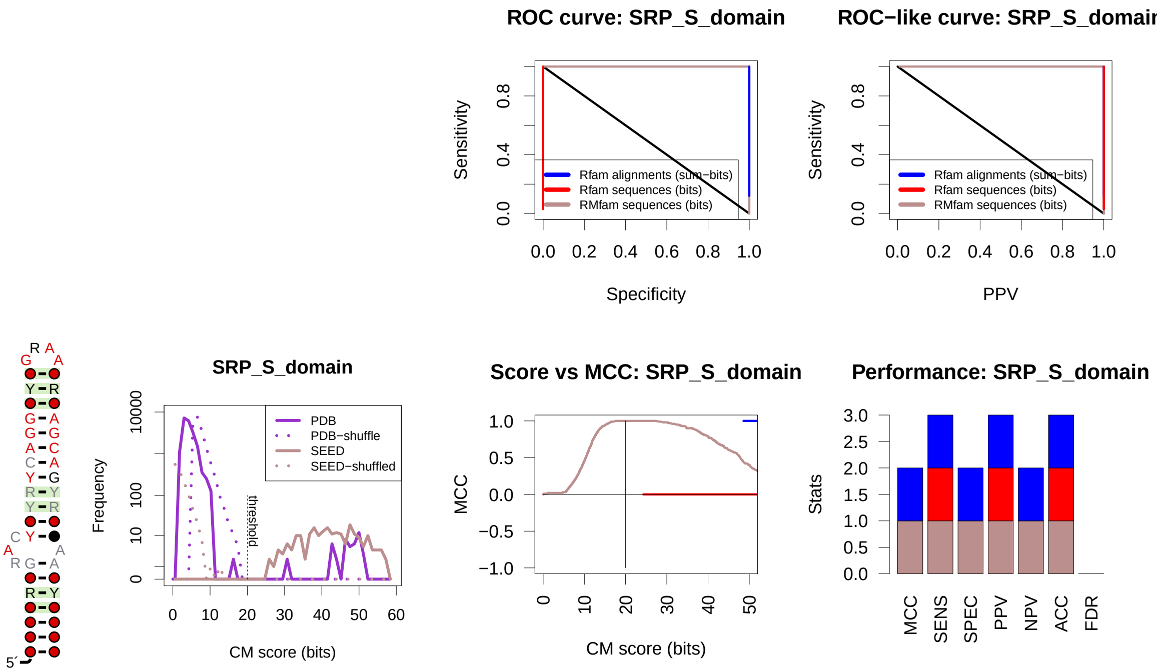

Figure 31. SRP_S_domain.

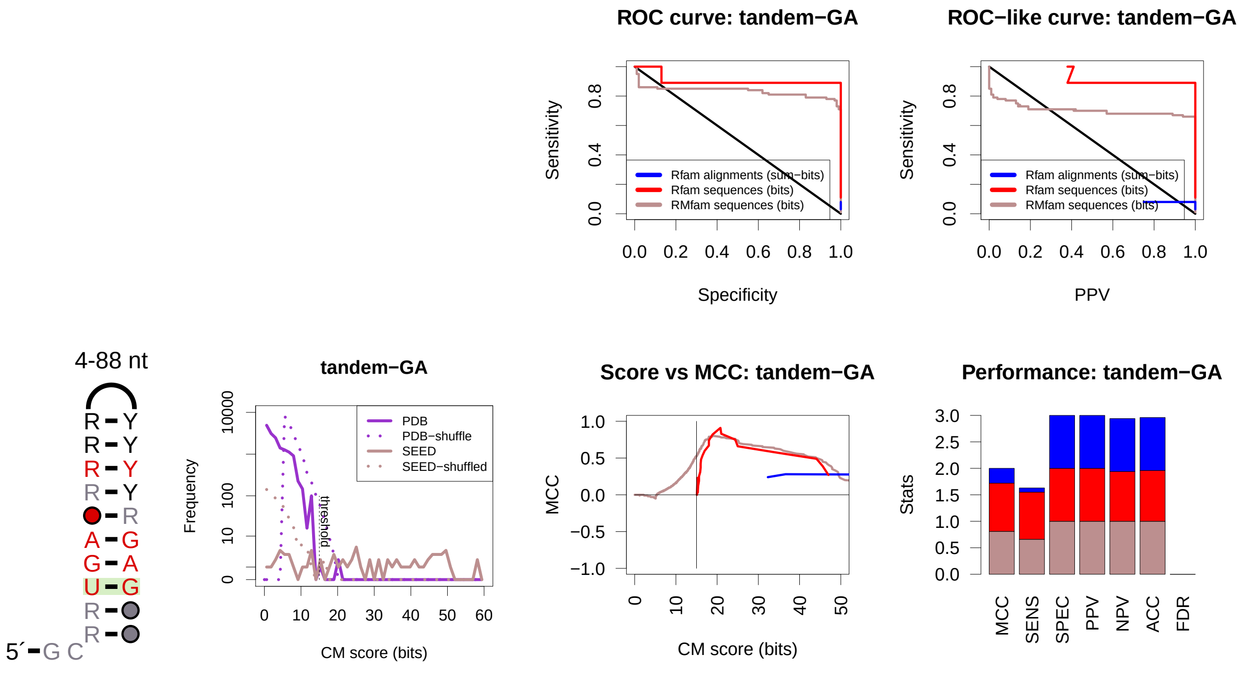

Figure 32. tandem-GA.

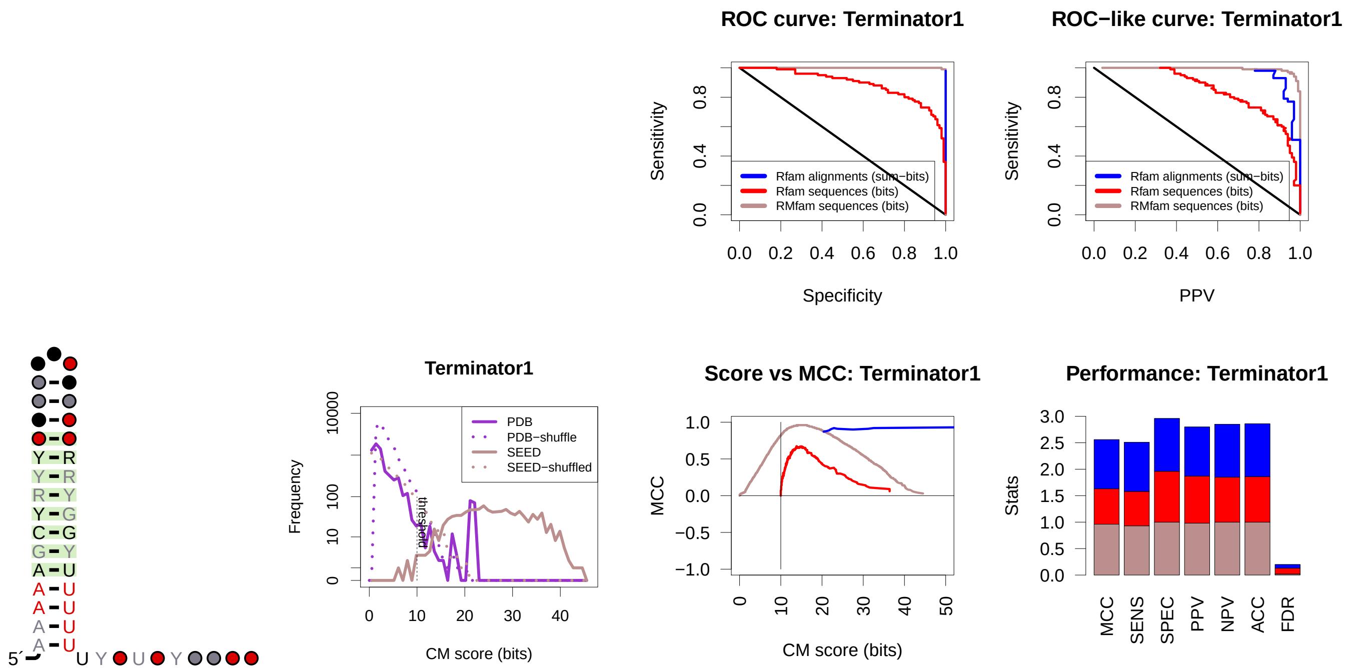

Figure 33. Terminator1.

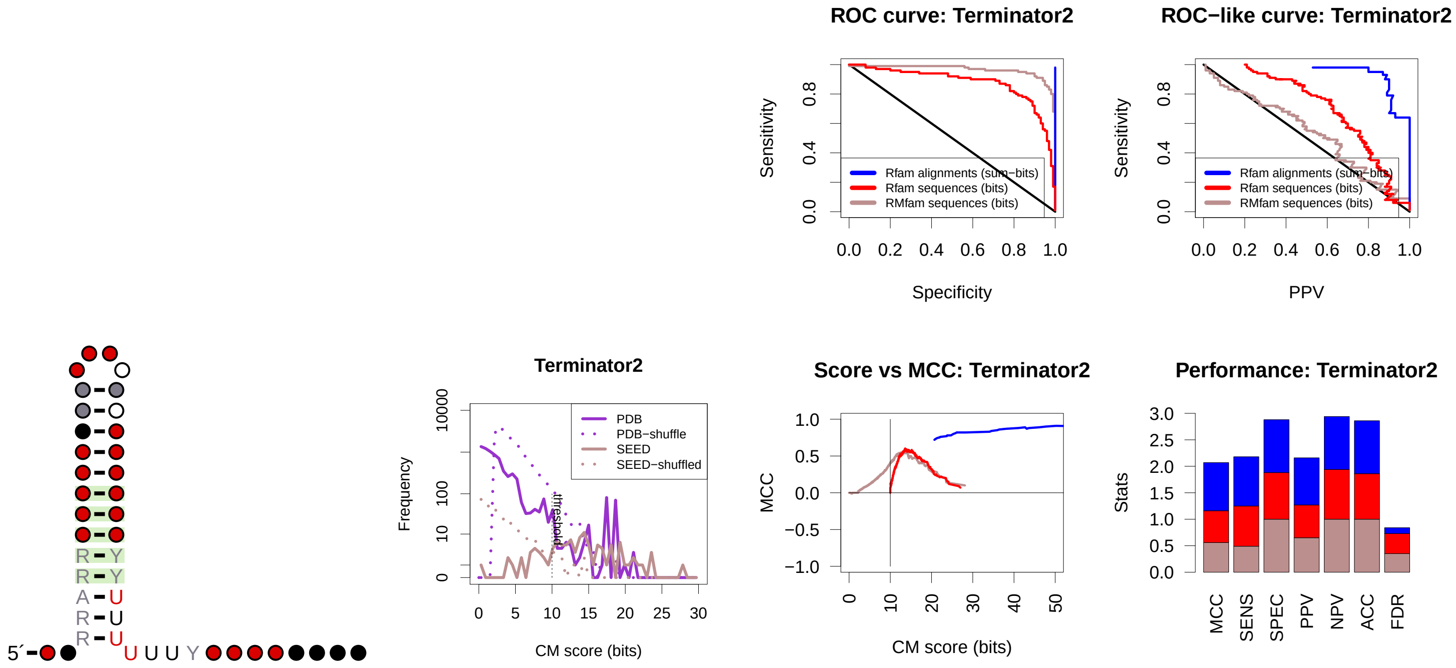

Figure 34. Terminator2.

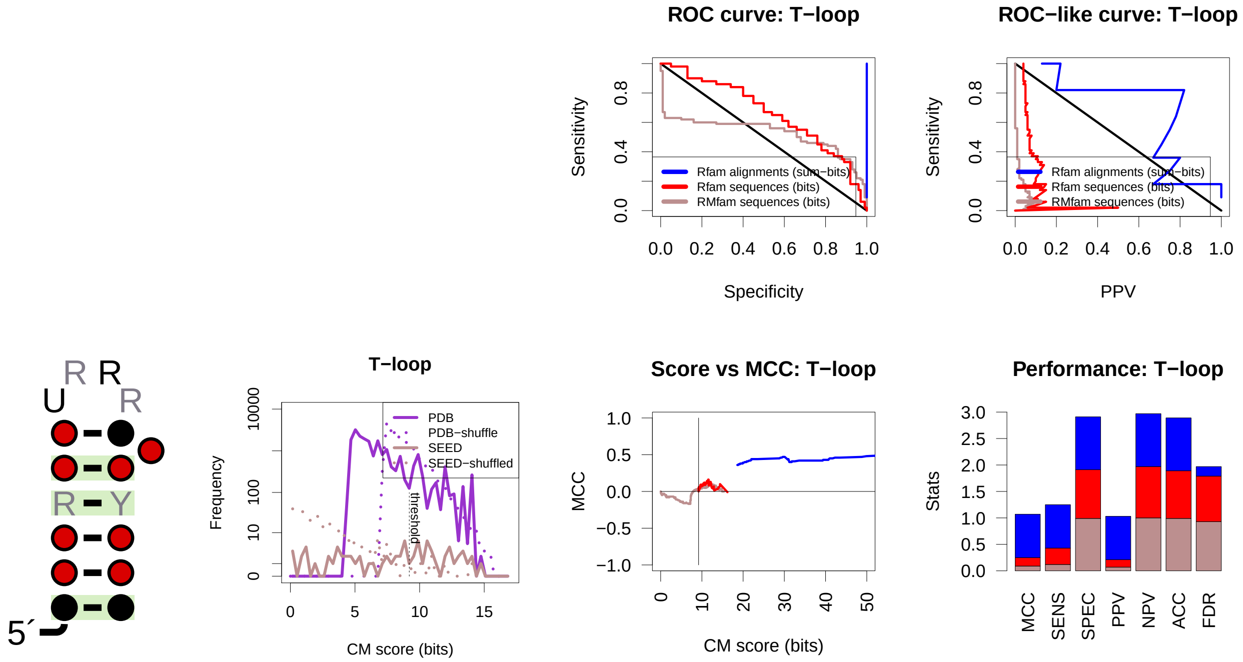

Figure 35. T-loop.

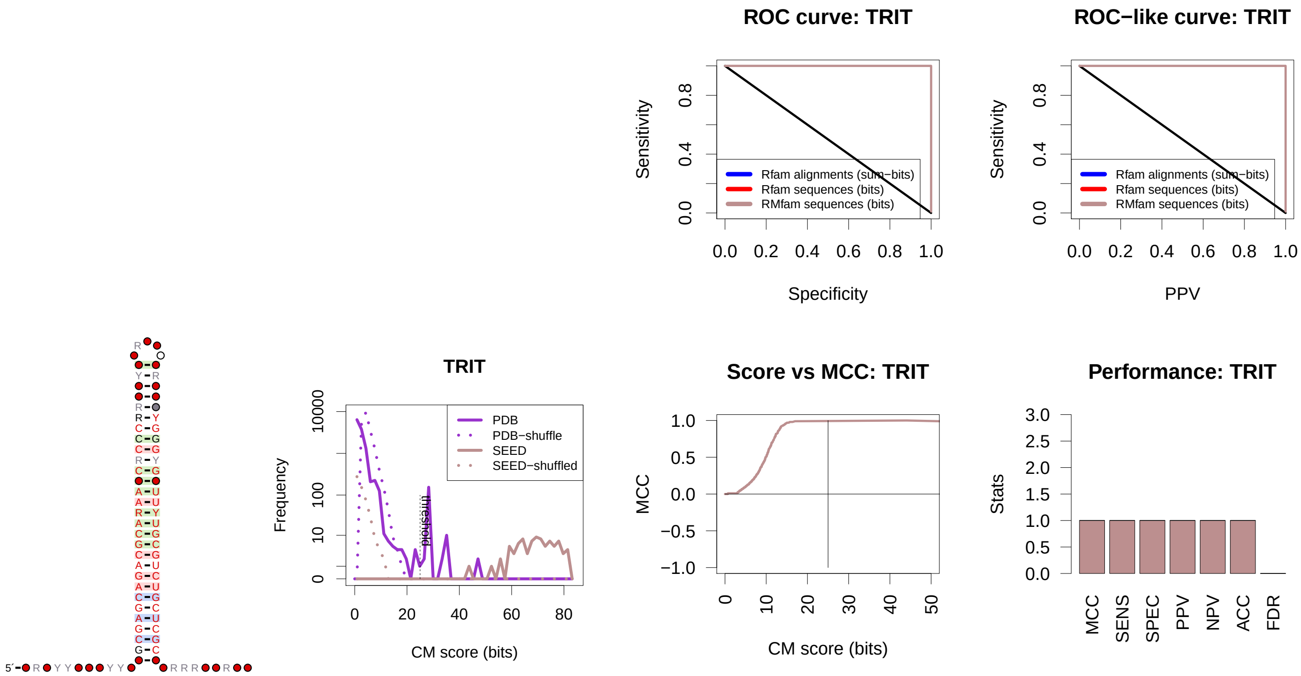

Figure 36. TRIT.

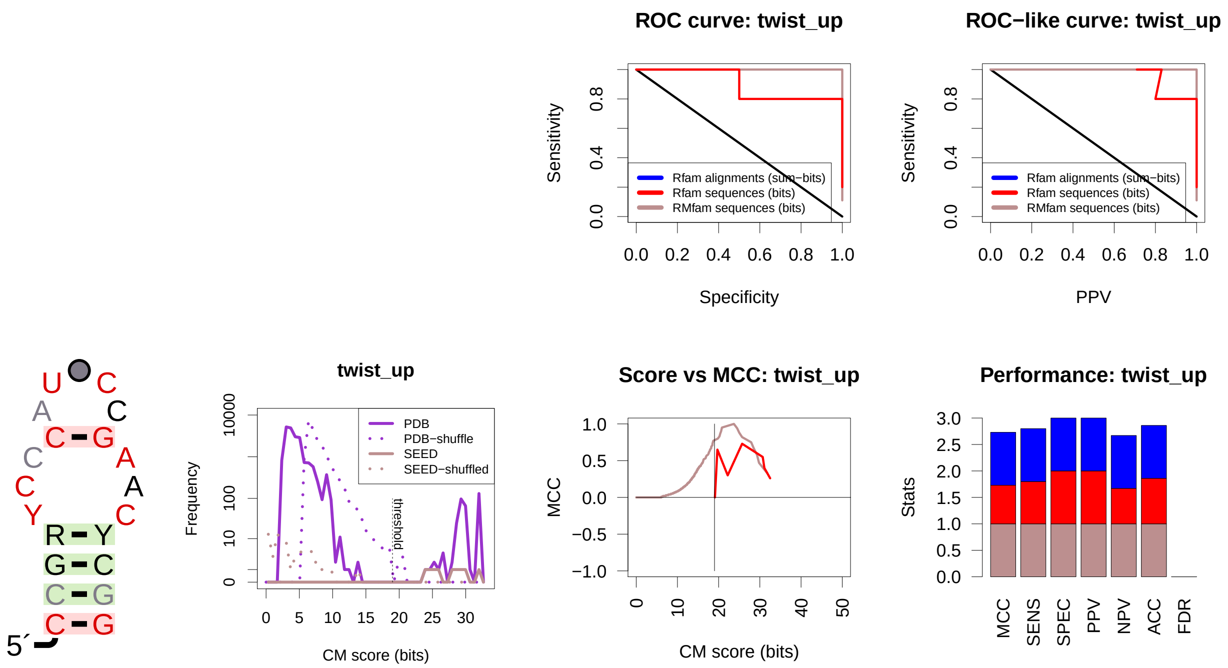

Figure 37. twist_up.

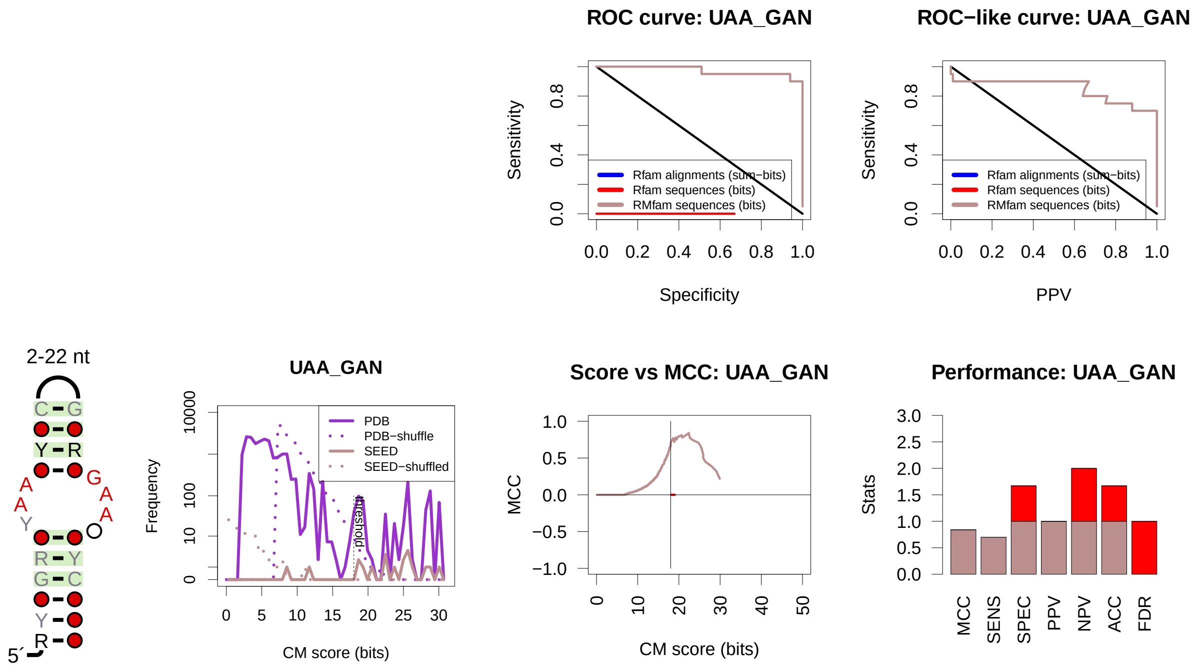

Figure 38. UAA_GAN.

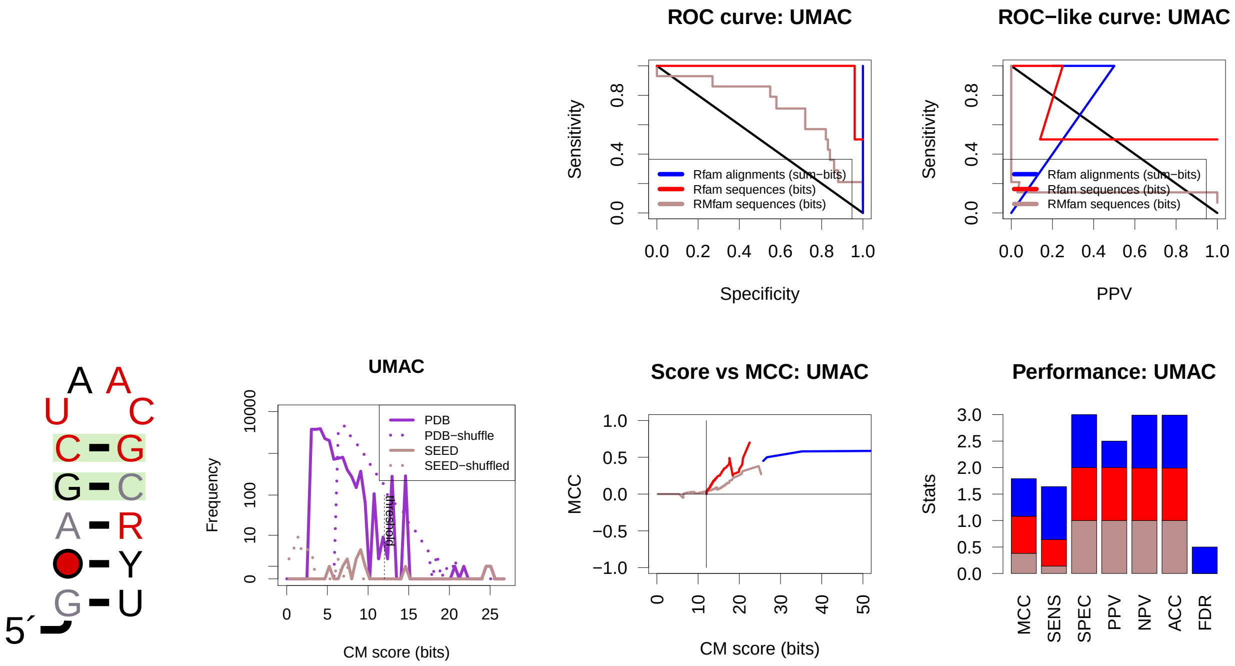

Figure 39. UMAC.

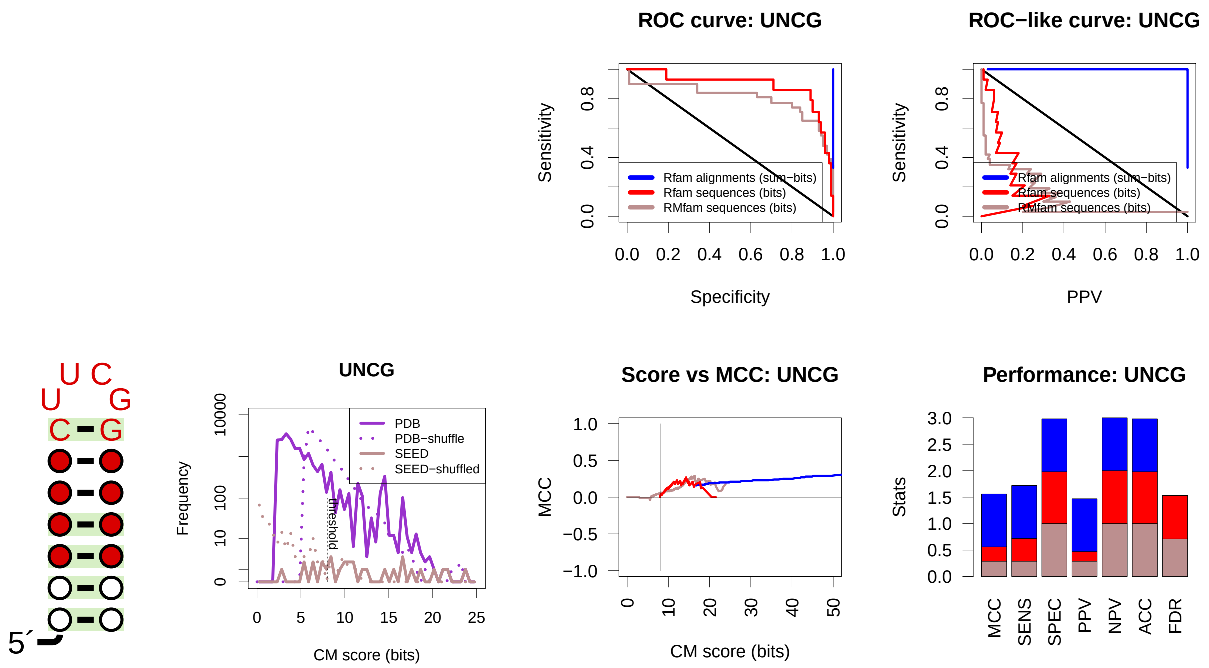

Figure 40. UNCG.

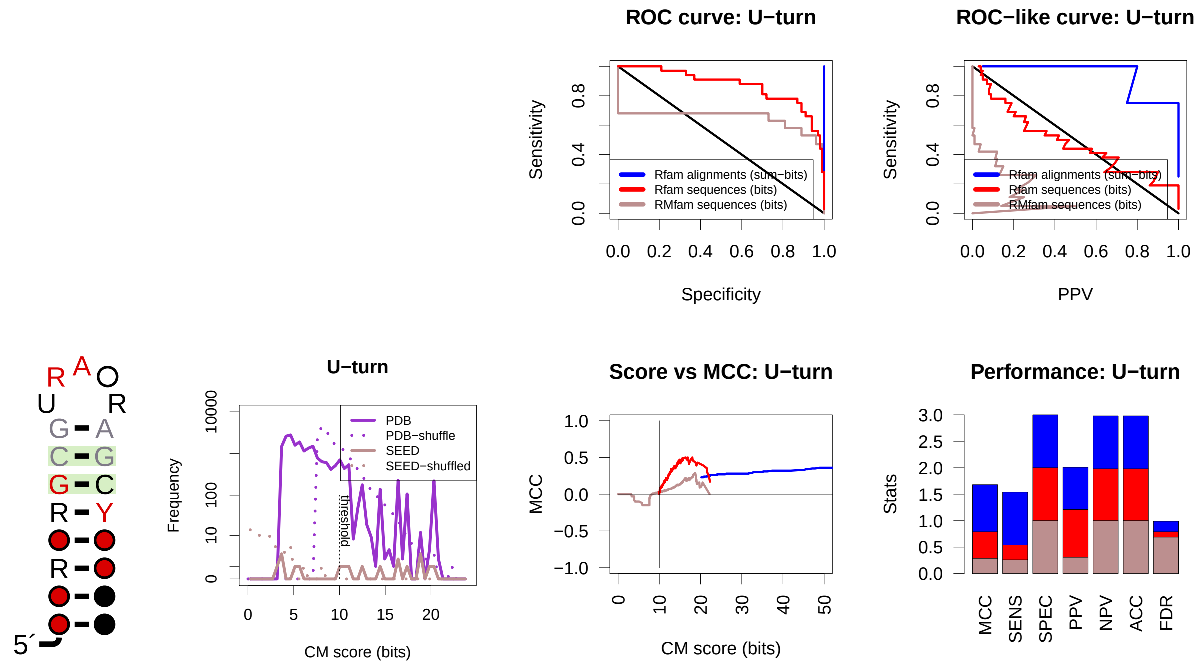

Figure 41. U-turn.

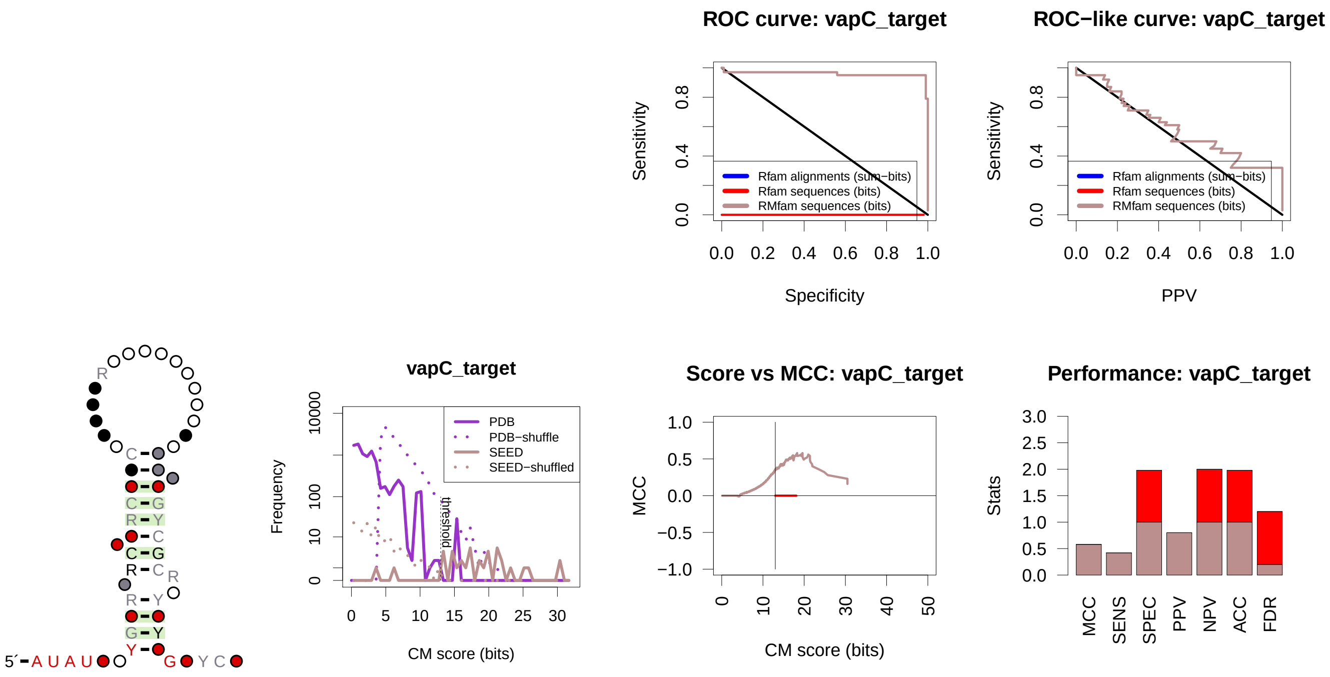

Figure 42. vapC_target.

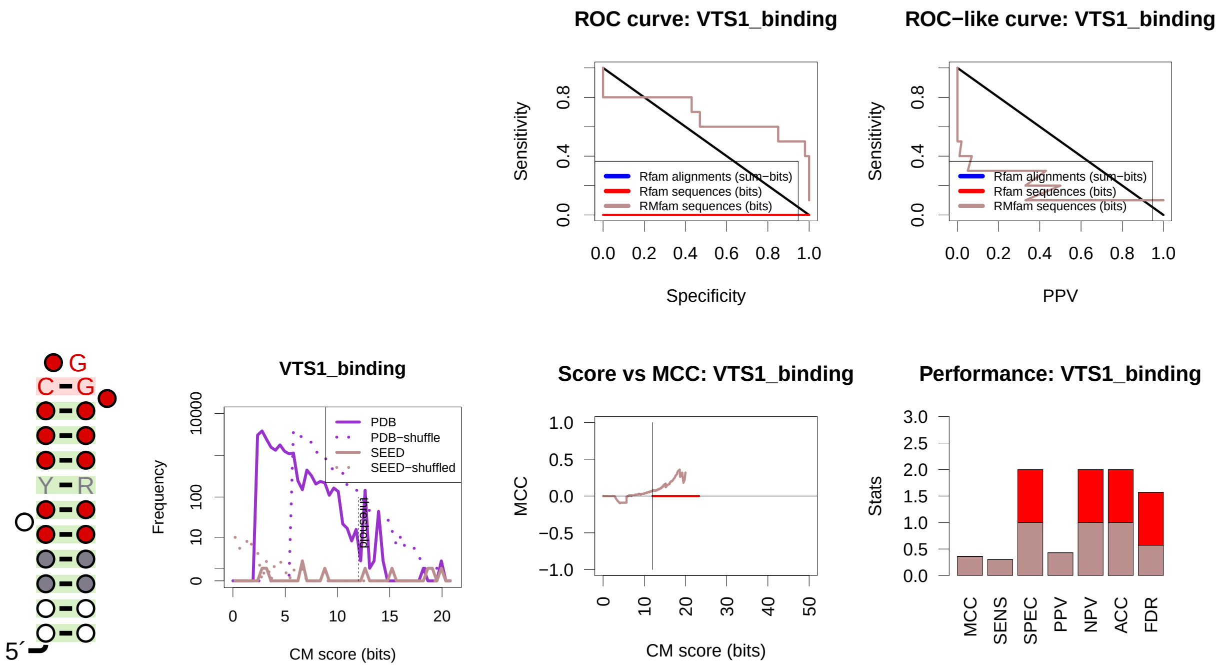

Figure 43. VTS1_binding.

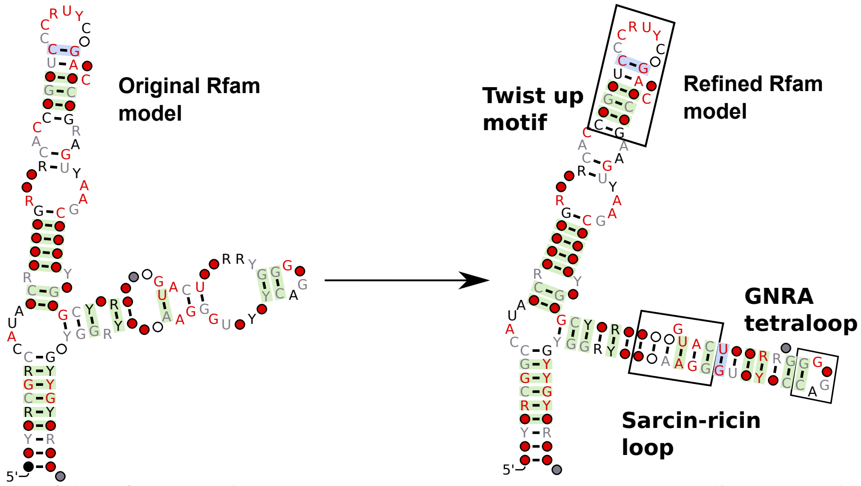

Figure 44. A comparison of the Rfam 11.0 5S rRNA consensus structure and a corresponding manually corrected model. The RMfam annotations identified a number of conserved motifs in the 5S rRNA model, using RMfam annotations as a guide. These include the twist up motif [17], a sarcin-ricin motif [50] and a GNRA motif [34]. The sarcin-ricin loop appeared to be mis-aligned in a number of cases in the Rfam alignment, the RMfam annotations allowed the alignment to be refined, correcting the alignment of this conserved motif.

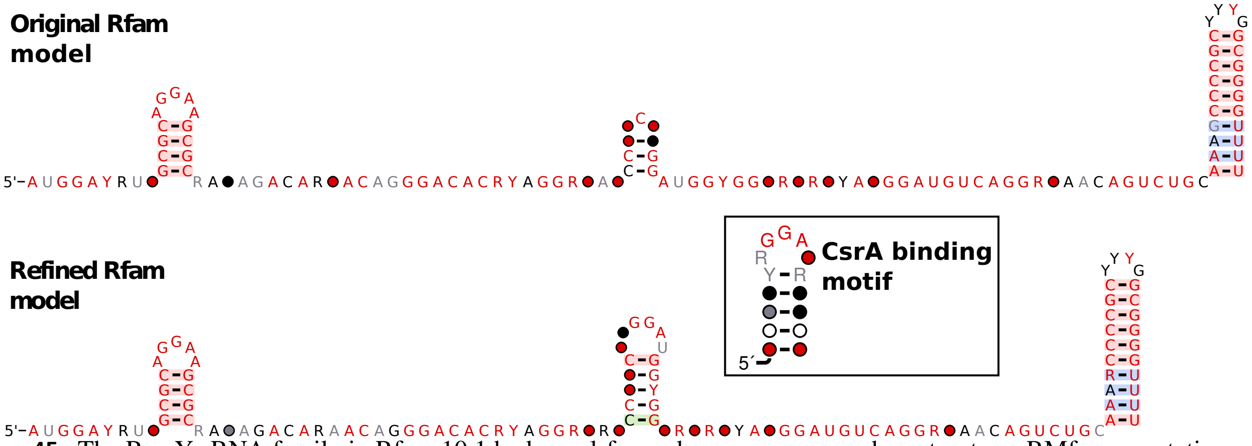

Figure 45. The RsmY sRNA family in Rfam 10.1 had a mal-formed consensus secondary structure. RMfam annotations identified an additional CsrA binding motif, which allowed the structure to be refined to emphasise this fact. CsrA is a dimeric protein that generally binds to two motifs, the refined structure has a better fit with this model [81, 82].

0.1 Networks

We can now gain insights into the network of RNA motifs and families. This reveals aspects of the evolutionary constraints on RNA structure as well as convergent evolution and function. An example of an extreme evolutionary constraint that we have observed is the GNRA tetraloop in the bacterial A, B and archaeal RNase P RNA families. This loop is located on the P9 helix of RNase P that appears to have been conserved throughout the evolutionary span of bacteria and archaea [4, 83]. The structurally diverse domains 1 - 4 for the group II introns families are also enriched with GNRA-tetraloop hosting helices near the 5′ end of the region (See Figure 46), other than this loop there is little that is conserved between these presumably homologous sequences and structures. A striking example of convergent evolution of analogous structures is the intrinsic bacterial transcription terminators [84] (See Figure S4). These motifs are required for the efficient termination of transcription [54]. We see that these are frequently used by many bacterial small RNAs and cis-regulatory elements such as 5′ leaders (See Figure 46), a result that serves to validate the accuracy of our method as well as illustrating the plasticity of transcription terminator evolution.

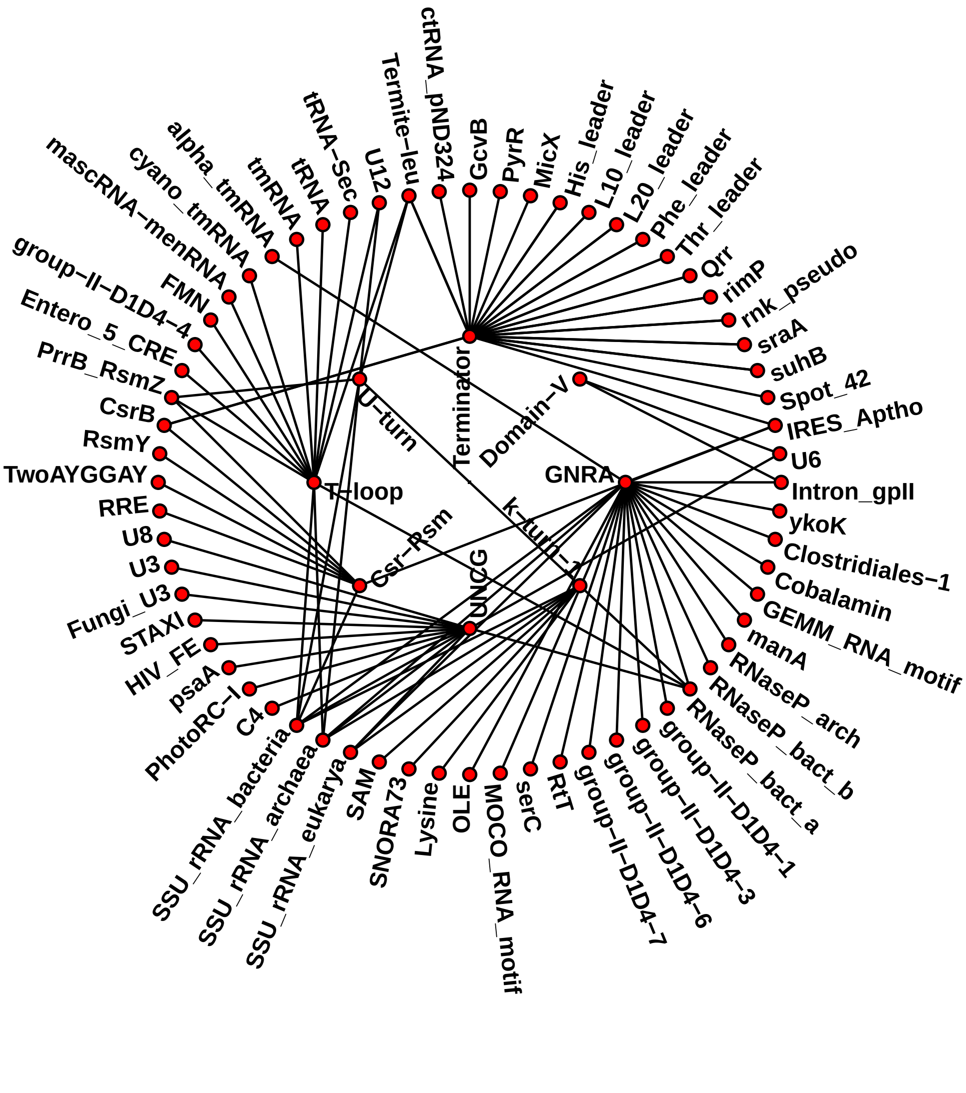

Figure 46. A network of the highest scoring 100 annotations of RMfam on Rfam. The nodes on the inner circle shows 8 RMfam motifs, the outer circle shows 64 Rfam families. The edges connecting the nodes indicate high-scoring predictions.

References

[1] P W Rose, C Bi, W F Bluhm, C H Christie, D Dimitropoulos, S Dutta, R K Green, D S Goodsell, A Prlic, M Quesada, G B Quinn, A G Ramos, J D Westbrook, J Young, C Zardecki, H M Berman, and P E Bourne. The rcsb protein data bank: new resources for research and education. *Nucleic Acids Res*, 41(Database issue):D475–82, Jan 2013.

[2] C Workman and A Krogh. No evidence that mrnas have lower folding free energies than random sequences with the same dinucleotide distribution. *Nucleic Acids Res*, 27(24):4816–22, Dec 1999.

[3] J A Cruz and E Westhof. Sequence-based identification of 3D structural modules in RNA with RMDetect. *Nat Methods*, 8(6):513–21, Jun 2011.

[4] J W Brown, J M Nolan, E S Haas, M A Rubio, F Major, and N R Pace. Comparative analysis of ribonuclease P RNA using gene sequences from natural microbial populations reveals tertiary structural elements. *Proc Natl Acad Sci U S A*, 93(7):3001–6, Apr 1996.

[5] D A Pomeranz Krummel and S Altman. Verification of phylogenetic predictions in vivo and the importance of the tetraloop motif in a catalytic rna. *Proc Natl Acad Sci U S A*, 96(20):11200–5, Sep 1999.

[6] S Nottrott, K Hartmuth, P Fabrizio, H Urlaub, I Vidovic, R Ficner, and R Lührmann. Functional interaction of a novel 15.5kd [u4/u6.u5] tri-snrnp protein with the 5' stem-loop of u4 snrna. *EMBO J*, 18(21):6119–33, Nov 1999.

[7] R R Gutell, J J Cannone, D Konings, and D Gautheret. Predicting U-turns in ribosomal RNA with comparative sequence analysis. *J Mol Biol*, 300(4):791–803, Jul 2000.

[8] E Ennifar, A Nikulin, S Tishchenko, A Serganov, N Nevskaya, M Garber, B Ehresmann, C Ehresmann, S Nikonov, and P Dumas. The crystal structure of uucg tetraloop. *J Mol Biol*, 304(1):35–42, Nov 2000.

[9] S Barends, K Björk, A P Gultyaev, M H de Smit, C W Pleij, and B Kraal. Functional evidence for d- and t-loop interactions in tmrna. *FEBS Lett*, 514(1):78–83, Mar 2002.

[10] A S Krasilnikov and A Mondragón. On the occurrence of the t-loop rna folding motif in large rna molecules. *RNA*, 9(6):640–3, Jun 2003.

[11] A S Krasilnikov, Y Xiao, T Pan, and A Mondragón. Basis for structural diversity in homologous rnas. *Science*, 306(5693):104–7, Oct 2004.

[12] S Nonin-Lecomte, B Felden, and F Dardel. Nmr structure of the aquifex aeolicus tmrna pseudoknot pk1: new insights into the recoding event of the ribosomal trans-translation. *Nucleic Acids Res*, 34(6):1847–53, 2006.

[13] E Sonnleitner, L Abdou, and D Haas. Small rna as global regulator of carbon catabolite repression in pseudomonas aeruginosa. *Proc Natl Acad Sci U S A*, 106(51):21866–71, Dec 2009.

[14] Z Weinberg, J X Wang, J Bogue, J Yang, K Corbino, R H Moy, and R R Breaker. Comparative genomics reveals 104 candidate structured RNAs from bacteria, archaea, and their metagenomes. *Genome Biol*, 11(3):R31, 2010.

[15] K T Schroeder, S A McPhee, J Ouellet, and D M Lilley. A structural database for k-turn motifs in RNA. *RNA*, 16(8):1463–8, Aug 2010.

[16] E R Lee, J L Baker, Z Weinberg, N Sudarsan, and R R Breaker. An allosteric self-splicing ribozyme triggered by a bacterial second messenger. *Science*, 329(5993):845–8, Aug 2010.

[17] C Zhong and S Zhang. Clustering RNA structural motifs in ribosomal RNAs using secondary structural alignment. *Nucleic Acids Res*, 40(3):1307–17, Feb 2012.

[18] W W Grabow, Z Zhuang, Z N Swank, J E Shea, and L Jaeger. The right angle (RA) motif: a prevalent ribosomal RNA structural pattern found in group I introns. *J Mol Biol*, 424(1-2):54–67, Nov 2012.

[19] J Lehmann, F Jossinet, and D Gautheret. A universal rna structural motif docking the elbow of trna in the ribosome, rnase p and t-box leaders. *Nucleic Acids Res*, 41(10):5494–502, May 2013.

[20] P P Gardner, A Wilm, and S Washietl. A benchmark of multiple sequence alignment programs upon structural RNAs. *Nucleic Acids Res*, 33(8):2433–2439, 2005.

[21] E K Freyhult, J P Bollback, and P P Gardner. Exploring genomic dark matter: a critical assessment of the performance of homology search methods on noncoding RNA. *Genome Res*, 17(1):117–125, Jan 2007.

[22] P Anandam, E Torarinsson, and W L Ruzzo. Multiperm: shuffling multiple sequence alignments while approximately preserving dinucleotide frequencies. *Bioinformatics*, 25(5):668–9, Mar 2009.

[23] T Gesell and S Washietl. Dinucleotide controlled null models for comparative rna gene prediction. *BMC Bioinformatics*, 9:248, 2008.

[24] Eric P Nawrocki and Sean R Eddy. *INFERNAL User's Guide*. HHMI Janelia, Janelia Farm Research Campus, Ashburn, USA, version 1.1 edition, 2013.

[25] S Washietl, I L Hofacker, and P F Stadler. Fast and reliable prediction of noncoding rnas. *Proc Natl Acad Sci U S A*, 102(7):2454–9, Feb 2005.

[26] M Gerstein, E L Sonnhammer, and C Chothia. Volume changes in protein evolution. *J Mol Biol*, 236(4):1067–78, Mar 1994.

[27] M A Convery, S Rowsell, N J Stonehouse, A D Ellington, I Hirao, J B Murray, D S Peabody, S E Phillips, and

P G Stockley. Crystal structure of an rna aptamer-protein complex at 2.8 a resolution. *Nat Struct Biol*, 5(2):133–9, Feb 1998.

[28] S Rowsell, N J Stonehouse, M A Convery, C J Adams, A D Ellington, I Hirao, D S Peabody, P G Stockley, and S E Phillips. Crystal structures of a series of rna aptamers complexed to the same protein target. *Nat Struct Biol*, 5(11):970–5, Nov 1998.

[29] P S Klosterman, D K Hendrix, M Tamura, S R Holbrook, and S E Brenner. Three-dimensional motifs from the SCOR, structural classification of RNA database: extruded strands, base triples, tetraloops and U-turns. *Nucleic Acids Res*, 32(8):2342–52, 2004.

[30] C R Woese, S Winker, and R R Gutell. Architecture of ribosomal rna: constraints on the sequence of "tetraloops". *Proc Natl Acad Sci U S A*, 87(21):8467–71, Nov 1990.

[31] J Wolters. The nature of preferred hairpin structures in 16s-like rrna variable regions. *Nucleic Acids Res*, 20(8):1843–50, Apr 1992.

[32] F M Jucker and A Pardi. Solution structure of the cuug hairpin loop: a novel rna tetraloop motif. *Biochemistry*, 34(44):14416–27, Nov 1995.

[33] V P Antao, S Y Lai, and I Tinoco. A thermodynamic study of unusually stable rna and dna hairpins. *Nucleic Acids Res*, 19(21):5901–5, Nov 1991.

[34] L Jaeger, F Michel, and E Westhof. Involvement of a gnra tetraloop in long-range rna tertiary interactions. *J Mol Biol*, 236(5):1271–6, Mar 1994.

[35] D L Abramovitz and A M Pyle. Remarkable morphological variability of a common rna folding motif: the gnra tetraloop-receptor interaction. *J Mol Biol*, 266(3):493–506, Feb 1997.

[36] N Leulliot, V Baumruk, M Abdelkafi, P Y Turpin, A Namane, C Gouyette, T Huynh-Dinh, and M Ghomi. Unusual nucleotide conformations in gnra and uncg type tetraloop hairpins: evidence from raman markers assignments. *Nucleic Acids Res*, 27(5):1398–404, Mar 1999.

[37] F M Jucker, H A Heus, P F Yip, E H Moors, and A Pardi. A network of heterogeneous hydrogen bonds in gnra tetraloops. *J Mol Biol*, 264(5):968–80, Dec 1996.

[38] M Sarver, C L Zirbel, J Stombaugh, A Mokdad, and N B Leontis. FR3D: finding local and composite recurrent structural motifs in RNA 3D structures. *J Math Biol*, 56(1-2):215–52, Jan 2008.

[39] C L Zirbel, J E Sponer, J Sponer, J Stombaugh, and N B Leontis. Classification and energetics of the base-phosphate interactions in RNA. *Nucleic Acids Res*, 37(15):4898–918, Aug 2009.

[40] Q Zhao, H C Huang, U Nagaswamy, Y Xia, X Gao, and G E Fox. Unac tetraloops: to what extent do they mimic gnra tetraloops? *Biopolymers*, 97(8):617–28, Aug 2012.

[41] J J Cannone, S Subramanian, M N Schnare, J R Collett, L M D'Souza, Y Du, B Feng, N Lin, L V Madabusi, K M Müller, N Pande, Z Shang, N Yu, and R R Gutell. The comparative RNA web (CRW) site: an online database of comparative sequence and structure information for ribosomal, intron, and other RNAs. *BMC Bioinformatics*, 3:2, 2002.

[42] M Molinaro and I Tinoco. Use of ultra stable uncg tetraloop hairpins to fold rna structures: thermodynamic and spectroscopic applications. *Nucleic Acids Res*, 23(15):3056–63, Aug 1995.

[43] A Lescoute, N B Leontis, C Massire, and E Westhof. Recurrent structural rna motifs, isostericity matrices and sequence alignments. *Nucleic Acids Res*, 33(8):2395–409, 2005.

[44] C Zhong, H Tang, and S Zhang. Rnamotifscan: automatic identification of rna structural motifs using secondary structural alignment. *Nucleic Acids Res*, 38(18):e176, Oct 2010.

[45] U Nagaswamy and G E Fox. Frequent occurrence of the t-loop rna folding motif in ribosomal rnas. *RNA*, 8(9):1112–9, Sep 2002.

[46] Z Zhuang, L Jaeger, and J E Shea. Probing the structural hierarchy and energy landscape of an rna t-loop hairpin. *Nucleic Acids Res*, 35(20):6995–7002, 2007.

[47] G J Quigley and A Rich. Structural domains of transfer rna molecules. *Science*, 194(4267):796–806, Nov 1976.

[48] S Blouin, R Chinnappan, and D A Lafontaine. Folding of the lysine riboswitch: importance of peripheral elements for transcriptional regulation. *Nucleic Acids Res*, 39(8):3373–87, Apr 2011.

[49] M Meyer, E Westhof, and B Masquida. A structural module in rnase p expands the variety of rna kinks. *RNA Biol*, 9(3), Mar 2012.

[50] N B Leontis and E Westhof. A common motif organizes the structure of multi-helix loops in 16 s and 23 s ribosomal rnas. *J Mol Biol*, 283(3):571–83, Oct 1998.

[51] C M Duarte, L M Wadley, and A M Pyle. Rna structure comparison, motif search and discovery using a reduced representation of rna conformational space. *Nucleic Acids Res*, 31(16):4755–61, Aug 2003.

[52] D Gautheret, D Konings, and R R Gutell. A major family of motifs involving G.A mismatches in ribosomal RNA. *J Mol Biol*, 242(1):1–8, Sep 1994.

[53] J C Lee, R R Gutell, and R Russell. The uaa/gan internal loop motif: a new rna structural element that forms a cross-strand aaa stack and long-range tertiary interactions. *J Mol Biol*, 360(5):978–88, Jul 2006.

[54] P P Gardner, L Barquist, A Bateman, E P Nawrocki, and Z Weinberg. RNIE: genome-wide prediction of bacterial intrinsic terminators. *Nucleic Acids Res*, 14(39):5845–5852, 2011.

[55] K Mazan-Mamczarz, Y Kuwano, M Zhan, E J White, J L Martindale, A Lal, and M Gorospe. Identification of a signature motif in target mrnas of rna-binding protein auf1. *Nucleic Acids Res*, 37(1):204–14, Jan 2009.

[56] M Y Liu, G Gui, B Wei, J F Preston, L Oakford, U Yüksel, D P Giedroc, and T Romeo. The rna molecule csrb binds to the global regulatory protein csra and antagonizes its activity in escherichia coli. *J Biol Chem*, 272(28):17502–10, Jul 1997.

[57] Y Cui, A Chatterjee, Y Liu, C K Dumenyo, and A K Chatterjee. Identification of a global repressor gene, rsma, of erwinia carotovora subsp. carotovora that controls extracellular enzymes, n-(3-oxohexanoyl)-l-homoserine lactone, and pathogenicity in soft-rotting erwinia spp. *J Bacteriol*, 177(17):5108–15, Sep 1995.

[58] T Weilbacher, K Suzuki, A K Dubey, X Wang, S Gudapaty, I Morozov, C S Baker, D Georgellis, P Babitzke, and T Romeo. A novel srna component of the carbon storage regulatory system of escherichia coli. *Mol Microbiol*, 48(3):657–70, May 2003.

[59] L Argaman, R Hershberg, J Vogel, G Bejerano, E G Wagner, H Margalit, and S Altuvia. Novel small rna-encoding genes in the intergenic regions of escherichia coli. *Curr Biol*, 11(12):941–50, Jun 2001.

[60] S Aarons, A Abbas, C Adams, A Fenton, and F O'Gara. A regulatory rna (prrb rna) modulates expression of secondary metabolite genes in pseudomonas fluorescens f113. *J Bacteriol*, 182(14):3913–9, Jul 2000.

[61] C Valverde, S Heeb, C Keel, and D Haas. Rsmy, a small regulatory rna, is required in concert with rsmz for gaca-dependent expression of biocontrol traits in pseudomonas fluorescens cha0. *Mol Microbiol*, 50(4):1361–79, Nov 2003.

[62] S Moll, D J Schneider, P Stodghill, C R Myers, S W Cartinhour, and M J Filiatrault. Construction of an rsmx co-variance model and identification of five rsmx non-coding rnas in pseudomonas syringae pv. tomato dc3000. *RNA Biol*, 7(5):508–16, 2010.

[63] P P Gardner, J Daub, J Tate, B L Moore, I H Osuch, S Griffiths-Jones, R D Finn, E P Nawrocki, D L Kolbe, S R Eddy, and A Bateman. Rfam: Wikipedia, clans and the decimal release. *Nucleic Acids Res*, 39(Database issue):D141–5, Jan 2011.

[64] I López de Silanes, M Zhan, A Lal, X Yang, and M Gorospe. Identification of a target RNA motif for RNA-binding protein HuR. *Proc Natl Acad Sci U S A*, 101(9):2987–92, Mar 2004.

[65] E Dassi, P Zuccotti, S Leo, A Provenzani, M Assfalg, M D'Onofrio, P Riva, and A Quattrone. Hyper conserved elements in vertebrate mrna 3'-utrs reveal a translational network of rna-binding proteins controlled by hur. *Nucleic Acids Res*, 41(5):3201–16, Mar 2013.

[66] K Leppek, J Schott, S Reitter, F Poetz, M C Hammond, and G Stoecklin. Roquin promotes constitutive mrna decay via a conserved class of stem-loop recognition motifs. *Cell*, 153(4):869–81, May 2013.

[67] T Aviv, A N Amborski, X S Zhao, J J Kwan, P E Johnson, F Sicheri, and L W Donaldson. The nmr and x-ray structures of the saccharomyces cerevisiae vts1 sam domain define a surface for the recognition of rna hairpins. *J Mol Biol*, 356(2):274–9, Feb 2006.

[68] T Aviv, Z Lin, G Ben-Ari, C A Smibert, and F Sicheri. Sequence-specific recognition of rna hairpins by the sam domain of vts1p. *Nat Struct Mol Biol*, 13(2):168–76, Feb 2006.

[69] D Ray, H Kazan, E T Chan, L Peña Castillo, S Chaudhry, S Talukder, B J Blencowe, Q Morris, and T R Hughes. Rapid and systematic analysis of the rna recognition specificities of rna-binding proteins. *Nat Biotechnol*, 27(7):667–70, Jul 2009.

[70] F C Oberstrass, A Lee, R Stefl, M Janis, G Chanfreau, and F H Allain. Shape-specific recognition in the structure of the vts1p sam domain with rna. *Nat Struct Mol Biol*, 13(2):160–7, Feb 2006.

[71] M J Filiatrault, P V Stodghill, J Wilson, B G Butcher, H Chen, C R Myers, and S W Cartinhour. Crcz and crcx regulate carbon source utilization in pseudomonas syringae pathovar tomato strain dc3000. *RNA Biol*, 10(2):245–55, Feb 2013.

[72] R Moreno, P Fonseca, and F Rojo. Two small rnas, crcy and crcz, act in concert to sequester the crc global regulator in pseudomonas putida, modulating catabolite repression. *Mol Microbiol*, 83(1):24–40, Jan 2012.

[73] J L McKenzie, J Robson, M Berney, T C Smith, A Ruthe, P P Gardner, V L Arcus, and G M Cook. A vapbc toxin-antitoxin module is a posttranscriptional regulator of metabolic flux in mycobacteria. *J Bacteriol*, 194(9):2189–204, May 2012.

[74] M Regalia, M A Rosenblad, and T Samuelsson. Prediction of signal recognition particle rna genes. *Nucleic Acids Res*, 30(15):3368–77, Aug 2002.

[75] M A Rosenblad, J Gorodkin, B Knudsen, C Zwieb, and T Samuelsson. Srpdb: Signal recognition particle database. *Nucleic Acids Res*, 31(1):363–4, Jan 2003.

[76] M A Rosenblad, N Larsen, T Samuelsson, and C Zwieb. Kinship in the SRP RNA family. *RNA Biol*, 6(5):508–16, 2009.

[77] M Seetharaman, N V Eldho, R A Padgett, and K T Dayie. Structure of a self-splicing group ii intron catalytic effector domain 5: parallels with spliceosomal u6 rna. *RNA*, 12(2):235–47, Feb 2006.

[78] S Valadkhan. Role of the snrnas in spliceosomal active site. *RNA Biol*, 7(3):345–53, 2010.

[79] J Shine and L Dalgarno. Determinant of cistron specificity in bacterial ribosomes. *Nature*, 254(5495):34–8, Mar 1975.

[80] Tom Fawcett. An introduction to ROC analysis. *Pattern recognition letters*, 27(8):861–874, 2006.

[81] O Duss, E Michel, M Yulikov, M Schubert, G Jeschke, and F H Allain. Structural basis of the non-coding RNA RsmZ acting as a protein sponge. *Nature*, 509(7502):588–92, May 2014.

[82] M Schubert, K Lapouge, O Duss, F C Oberstrass, I Jelesarov, D Haas, and F H Allain. Molecular basis of messenger RNA recognition by the specific bacterial repressing clamp RsmA/CsrA. *Nat Struct Mol Biol*, 14(9):807–13, Sep 2007.

[83] J K Harris, E S Haas, D Williams, D N Frank, and J W Brown. New insight into RNase P RNA structure from comparative analysis of the archaeal RNA. *RNA*, 7(2):220–32, Feb 2001.

[84] M Naville and D Gautheret. Transcription attenuation in bacteria: theme and variations. *Brief Funct Genomic Proteomic*, 8(6):482–92, Nov 2009.
